# Placental plasminogen activator inhibitor 1 is induced by platelet-derived TGF-β independently of TGF-β receptor 3 and is upregulated in preeclampsia

**DOI:** 10.1093/molehr/gaag040

**Published:** 2026-06-29

**Authors:** Désirée Forstner, Azra Kulovic-Sissawo, Freya Lyssy, Jacqueline Guettler, Djenana Vejzovic, Beate Rinner, Daniel Kummer, Nadja Kupper, Christina Stern, Michael Gruber, Lena Neuper, Christine Daxboeck, Anubhuti Gupta, Shrey Kohli, Berend Isermann, Martin Gauster

**Affiliations:** Division of Cell Biology, Histology and Embryology, Gottfried Schatz Research Center, Medical University of Graz, Graz, Austria; Department of Obstetrics and Gynaecology, Medical University of Graz, Graz, Austria; Division of Cell Biology, Histology and Embryology, Gottfried Schatz Research Center, Medical University of Graz, Graz, Austria; Division of Cell Biology, Histology and Embryology, Gottfried Schatz Research Center, Medical University of Graz, Graz, Austria; Diagnostic and Research Institute of Pathology, Medical University of Graz, Graz, Austria; Diagnostic and Research Institute of Pathology, Medical University of Graz, Graz, Austria; Division of Cell Biology, Histology and Embryology, Gottfried Schatz Research Center, Medical University of Graz, Graz, Austria; Division of Cell Biology, Histology and Embryology, Gottfried Schatz Research Center, Medical University of Graz, Graz, Austria; Department of Obstetrics and Gynaecology, Medical University of Graz, Graz, Austria; Division of Cell Biology, Histology and Embryology, Gottfried Schatz Research Center, Medical University of Graz, Graz, Austria; Division of Cell Biology, Histology and Embryology, Gottfried Schatz Research Center, Medical University of Graz, Graz, Austria; Division of Cell Biology, Histology and Embryology, Gottfried Schatz Research Center, Medical University of Graz, Graz, Austria; Institute of Laboratory Medicine, Clinical Chemistry, and Molecular Diagnostics, University Hospital Leipzig, Leipzig University, Leipzig, Germany; Institute of Laboratory Medicine, Clinical Chemistry, and Molecular Diagnostics, University Hospital Leipzig, Leipzig University, Leipzig, Germany; Leipzig Reproductive Health Research Center (LE-REP), Leipzig University, Leipzig, Germany; Institute of Laboratory Medicine, Clinical Chemistry, and Molecular Diagnostics, University Hospital Leipzig, Leipzig University, Leipzig, Germany; Leipzig Reproductive Health Research Center (LE-REP), Leipzig University, Leipzig, Germany; Division of Cell Biology, Histology and Embryology, Gottfried Schatz Research Center, Medical University of Graz, Graz, Austria

**Keywords:** pregnancy, preeclampsia, trophoblast, platelet-derived factors, PAI-1, fibrin, TGF-β signaling

## Abstract

Preeclampsia is a pregnancy complication associated with *de novo* gestational hypertension, proteinuria, and dysregulations in the coagulation and fibrinolytic systems. Platelet-derived factors include various growth and differentiation factors, such as transforming growth factor (TGF)-β. The TGF-β type III receptor (TGFBR3), also known as betaglycan, is a co-receptor for the TGF-β superfamily and an important regulator of reproduction and fetal development. Here, we test the hypothesis whether or not TGFBR3 is involved in platelet-mediated upregulation of placental plasminogen activator inhibitor-1 (PAI-1). Perivillous fibrin deposits and PAI-1 levels were analyzed via immunohistochemistry, gene expression as well as protein expression in healthy and preeclamptic placental tissue. Placental villi and trophoblast cell line BeWo were incubated in presence or absence of washed platelets from pregnant women, platelet-derived soluble factors, or platelet-derived extracellular vesicles. Additionally, BeWo cells were treated with TGF-β-receptor inhibitors and silenced for TGFBR3 expression.

PAI-1 and its encoding gene *SERPINE1*, as well as perivillous fibrin deposits, were significantly increased towards term of gestation and showed a significant upregulation in placental villi from preeclamptic pregnancies. Furthermore, *SERPINE1* and PAI-1 were substantially upregulated in response to washed platelets and platelet-derived factors, but not to platelet-derived extracellular vesicles. The upregulation of *SERPINE1* was blocked to control levels with the TGF-β inhibitor P144, whereas silencing of TGFBR3 had no effect.

Our results suggest that platelet-derived factors induce placental PAI-1 expression independently of TGF-β receptor 3. Increased maternal platelet activation may contribute to significantly upregulated placental PAI-1 and perivillous fibrin deposits in preeclampsia.

## Introduction

Hypertensive disorders during pregnancy are a major contributor to both maternal and infant health problems and deaths worldwide. Among hypertensive disorders of pregnancy, preeclampsia (PE) is the most dangerous variant. The precise etiology of PE remains incompletely understood, but there is a common consensus that it arises from a combination of multiple factors, rendering it a multifactorial disease. Among the factors associated with the manifestation of PE are abnormalities of the coagulation and fibrinolytic system (Pinheiro *et al.*, 2013; Ye *et al.*, 2017; Kobayashi *et al.*, 2023). While increases in coagulation factor levels and decreases in natural anticoagulation and fibrinolytic activity results in an overall hypercoagulable state in physiological normotensive pregnancies, PE is accompanied by an even greater increase in hypercoagulability.

A key player of the fibrinolytic system is plasminogen, which needs to be converted from its inactive precursor variant into active plasmin by urokinase-type plasminogen activator (uPA), tissue-type plasminogen activator (tPA), kallikrein and factor XII (Hageman factor, *F12*) (Chapin and Hajjar, 2015). Plasmin drives fibrinolysis by degradation of fibrin meshworks that had been generated by thrombin-mediated conversion of fibrinogen into fibrin and subsequent cross-linking by Factor XIII (Bagoly *et al.*, 2012). There are two ways how the action of plasmin is suppressed, including direct inhibition by alpha 2-antiplasmin (encoded by *SERPINF2*) and alpha-2-macroglobulin (*A2M*) or indirectly by inhibition of its conversion from the zymogen into its active variant by plasminogen activator inhibitors type 1 (PAI-1, *SERPINE1*), type 2 (PAI-2, *SERPINB2*), and type 3 (PAI-3, *SERPINA3*) as well as C1-esterase inhibitor (*SERPING1*) and protease nexin-1 (PN-1, *SERPINE2*) (Al-Horani, 2014). These plasminogen activator inhibitors act by inhibiting uPA and tPA without directly inhibiting plasmin. PAI-1 is considered the major inhibitor of the fibrinolytic system, accounting for approximately sixty percent of the plasminogen activator inhibitor activity and by far exceeding that of PAI-2 and -3 during pregnancy (Ye *et al.*, 2017). Circulating PAI-1 is mainly stored in platelet α-granules, with additional expression in endothelial and smooth muscle cells, adipocytes, and hepatocytes (Morrow and Mutch, 2023).

When it comes to pregnancy, the epithelial-like trophoblast layer, consisting of mononucleated cytotrophoblasts and the overlying multinucleated syncytiotrophoblast that lines placental villi, represents another source for PAI-1 (Hofmann *et al.*, 1994). PAI-1 plasma levels start to rise with the second trimester of healthy human pregnancy, peaking at 32–40 weeks’ gestation, followed by a decline to levels comparable with levels before pregnancy within 6 weeks postpartum (Choi and Pai, 2002; Hellgren, 2003). Several studies have shown elevated plasma levels of PAI-1 in women with PE compared to healthy controls; however, it is worth noting that some studies have reported no significant difference (Agersnap *et al.*, 2022), warranting further investigation. Increased proinflammatory IL-1β and/or hypoxic conditions, as suggested for PE, can induce trophoblastic PAI-1 expression (Fitzpatrick and Graham, 1998; Prutsch *et al.*, 2012) that in turn could lead to increased perivillous fibrin deposition, damage of the syncytial layer and reduced nutrient transfer. Another key regulator is TGF-β, which induces PAI-1 in trophoblasts through SMAD transcription factor-dependent pathways and the co-activators CBP and/or p300 (Dennler *et al.*, 1998; Forstner *et al.*, 2020). TGF-β initiates the signaling cascade upon binding to the transmembrane TGF-β receptor 2 (*TGFBR2*), which is followed by recruitment and activation of TGF-β receptor 1 (*TGFBR1*), resulting in an active heteromeric receptor complex that activates SMAD proteins (Vander Ark *et al.*, 2018). TGF-β receptor 3 (*TGFBR3*), also referred to as betaglycan, enhances cellular responsiveness to TGF-β2 and acts as a co-receptor that promotes signaling complex assembly (Kim *et al.*, 2019). Knowledge on spatial expression of *TGFBR3* in the human placenta is limited (Ciarmela *et al.*, 2003), and its potential role in regulating placental PAI-1 expression has not been addressed so far. In this study, we aimed to address the hypothesis that maternal platelet activation and release of their cargo at the maternal–fetal interface induces placental PAI-1 production via TGF-β receptor 3.

## Materials and methods

### Placental tissue collection

The study was approved by the ethical committee of the Medical University of Graz (31-019 ex 18/19). Human first trimester placental tissue with gestational age (GA) between week 5 and 12 was collected with written informed consent from women undergoing legal elective surgical pregnancy terminations at a local gynecologist (Femina Med). Pre-term (GA 34 + 0 to 37 + 0) and term (GA 37 + 0 to 41 + 0) placental tissues were obtained with written informed consent at the Department of Obstetrics and Gynecology at the Medical University of Graz mainly after a caesarean section. PE was diagnosed, classified, and managed in accordance with the guideline “Hypertensive Disorders in Pregnancy (HDP): Diagnostics and Therapy (DGGG, OEGGG and SGGG, S2k-Level,” AWMF Registry No. 015/018, June 2024) (Pecks *et al.*, 2025). Women were included in the PE cohort if they met these diagnostic criteria, which align with the international recognized guidelines of the ISSHP (Magee *et al.*, 2022), the ACOG (Gestational hypertension and preeclampsia: ACOG Practice Bulletin, Number 222, 2020) and the NICE (Hypertension in Pregnancy: Diagnosis and Management, 2019), defined as the new-onset of hypertension after 20 weeks of gestation (systolic blood pressure ≥140 mmHg and/or diastolic blood pressure ≥90 mmHg on at least two occasions) in combination with proteinuria (≥300 mg/24 h or protein/creatinine ratio ≥0.3) and/or evidence of maternal organ or uteroplacental dysfunction. For subgroup analyses, late-onset preeclampsia (loPE) was categorized based on the gestational age at diagnosis (Robillard *et al.*, 2020; Masini *et al.*, 2022), with loPE defined as a diagnosis occurring at or beyond 34 + 0 weeks. Placental villous tissue was rinsed in buffered saline and dissected into small pieces before either performing *ex vivo* experiments or snap freezing in liquid nitrogen until further processing. Patient characteristics of the study groups are summarized in Supplementary Table S1.

### Placental villous explant culture

Placental villous explants with a size of approximately 1–2 mm (Forstner *et al.*, 2020), were placed into 24-well dishes (Nunc, Thermo Fisher Scientific, Roskilde, Denmark) and cultured in 1 ml/well of DMEM/F12 (1:1, Gibco^TM^, Thermo Fisher Scientific, Waltham, MA, USA) supplemented with 10% v/v fetal bovine serum (FBS) (Gibco^TM^, Thermo Fisher Scientific), 0.1 U/ml Penicillin/Streptomycin (Gibco^TM^, Thermo Fisher Scientific) and 1% v/v L-glutamine (Gibco^TM^, Thermo Fisher Scientific) in a hypoxic workstation (BioSpherix LtD; Redfield, NY, USA) under a humidified atmosphere at 5% CO_2_ and 37**°**C. First trimester placental explants were cultured under 2.5% O_2_, while term tissue was cultured under 8% O_2_ for 24 h. Patient characteristics of the study group are summarized in Supplementary Table S2.

### Isolation of human platelets from whole blood samples and preparation of platelet releasate

Citrated whole blood samples (Vacuette^®^, Greiner Bio-One GmbH, Kremsmünster, Austria) from healthy pregnant donors with gestational age between 34 and 41 weeks were obtained at the Department of Obstetrics and Gynecology, Medical University of Graz, with written informed consent. Platelets were isolated as previously described (Forstner *et al.*, 2023; Lyssy *et al.*, 2025) and a detailed description is provided in the Supplementary Materials and Methods. After the platelet isolation, washed platelets were activated with 1 U/ml thrombin (Sigma-Aldrich, St. Louis, MO, USA) for at least 30 min, and subsequently thrombin was inactivated with 1.1 U/ml hirudin (Merck KGaA, Darmstadt, Germany). Platelets were then pelleted by centrifugation at 1962 g for 15 min at room temperature (RT) and finally the supernatant, representing the so-called platelet releasate (PR), was collected and stored at −80**°**C until further use. Patient characteristics of the study group used for the preparation of platelet releasate are summarized in Supplementary Table S3.

### Inhibitor treatments of trophoblast cells

BeWo cells were seeded with a density of 1 × 10^5^/ml one day prior to the differentiation into a multinucleated syncytium with forskolin (20 µM; Bio-Techne, Tocris, Abingdon, UK) for 48 h, and afterwards cells were preincubated with 10 µM TGF-β R1 inhibitor SB431542 (Sigma-Aldrich) for 2 h. After pre-incubation, medium was exchanged with fresh medium containing either SB431542 (Sigma-Aldrich) inhibitor and/or PR for 24 h.

TGF-β1 inhibitor peptide 144 (Disetertide^©^, Biomatik, Kitchener, ON, Canada), derived from the sequence of the extracellular region of TGF-β type III receptor (Betaglycan), was preincubated with 10% v/v pooled human platelet lysate (pHPL) (Schallmoser and Strunk, 2009; Forstner *et al.*, 2020) for 1 h at RT under gentle movement. Afterwards, BeWo cells were stimulated with either forskolin (20 µM; Bio-Techne, Tocris) or vehicle control DMSO (0.1% v/v) in the presence or absence of 10% v/v pHPL in addition to P144 in a final concentration of 10 µg/ml and 100 µg/ml for 24 h. Culture medium was supplemented with heparin (Merck KGaA, Darmstadt, Germany) in a final concentration of 2 U/ml, to prevent coagulation.

### Stimulation with TGFβ1

BeWo cells were seeded with a density of 1 × 10^5^ cells/ml in DMEM/F12 supplemented with 10% v/v FBS (Gibco^TM^, Thermo Fisher Scientific), 0.1 U/ml Penicillin/Streptomycin (Gibco^TM^, Thermo Fisher Scientific), and 1% v/v L-glutamine (Gibco^TM^, Thermo Fisher Scientific) and incubated overnight in a humidified atmosphere of 5% CO_2_ at 37°C. Afterwards, cells were either treated with human recombinant TGF-β1 (R&D Systems, Inc., Minneapolis, MN, USA) at a final concentration of 20 ng/ml or the solvent control (4 mM HCl-solution supplemented with 1 mg/ml BSA) for 24 h.

### Silencing of TGFBR1 and TGFBR3 in trophoblast cells

BeWo cells were seeded in 24-well dishes in DMEM/F-12 media supplemented with 10% v/v FBS (Gibco^TM^, Thermo Fisher Scientific), 0.1 U/ml Penicillin/Streptomycin (Gibco^TM^, Thermo Fisher Scientific), and 1% v/v L-glutamine (Gibco^TM^, Thermo Fisher Scientific) until they reached a confluency of approximately 80%. Afterwards, cells were transfected with pre-designed small interfering RNA (siRNA) prior to treatment with forskolin (Bio-Techne, Tocris) and pHPL. Cells were transfected with TGFBR1 or TGFBR3 Silencer^®^ Select pre-designed siRNA (2.5 and 5 pmol; Ambion^®^, Thermo Fisher Scientific, Waltham, MA, USA) using Lipofectamine^TM^ RNAiMAX Transfection Reagent (Invitrogen^TM^ Corporation, Thermo Fisher Scientific, Carlsbad, CA, USA). After preincubation of the siRNA with Lipofectamine for 5 min, cells were transfected for 24 h in DMEM/F12 media supplemented with 1% v/v L-glutamine (Gibco^TM^, Thermo Fisher Scientific). Subsequently, the cells were treated with 20 µM forskolin (Bio-Techne, Tocris), or 0.1% v/v DMSO (Carl Roth, Karlsruhe, Germany) as solvent control, in the presence or absence of 10% v/v pHPL and heparin (Merck KGaA) at a final concentration of 2 IU/ml for 24 h.

### Co-culture of trophoblast cells with platelet-derived factors and washed platelets

BeWo cells were seeded with a density of 1 × 10^5^/ml and differentiated with forskolin (20 µM; Bio-Techne, Tocris) for 48 h. Subsequently, five different treatments were started for 24 h. For treatment with platelet lysate, pHPL, which was kindly provided by the Department of Transfusion Medicine at the Paracelsus Medical University of Salzburg, was produced as previously described (Schallmoser and Strunk, 2009). Cells were treated with media supplemented with or without 10% v/v pHPL and heparin (Merck KGaA) at a final concentration of 2 IU/ml. For treatment with platelet-derived factors, thrombin-derived PR, pooled from individual donors and subsequently mixed 1:2 with media, was used. Cells were also treated with 1 IU/ml thrombin (Sigma-Aldrich) alone, which served as a control. For co-culture with platelets, washed platelets from healthy pregnant women were either directly administered to the trophoblast cell layer or transferred into a polycarbonate cell culture insert with a pore size of 0.4 µm (Nunc Lab-Tek, Thermo Scientific, Waltham, MA, USA) as previously described (Lyssy *et al.*, 2023), to prevent direct cell contact between platelets and trophoblast cells. Platelets were then activated with 1 IU/ml thrombin (Sigma-Aldrich), and after 24 h of treatment, cell lysates were collected.

### Incubation of placental explants with washed platelets and platelet-derived factors

As previously described, term placental villi were dissected into small pieces and subsequently incubated with washed platelets, which were activated with 1 IU/ml thrombin (Sigma-Aldrich). Of note, washed platelets were isolated from the same patient from whom the placenta samples were obtained for each experiment. For incubation with platelet-derived factors, the PR of at least 10 female donors was pooled and diluted 1:2 with culture medium containing a final concentration of 10% v/v FBS (Gibco^TM^, Thermo Fisher Scientific). Explants were incubated in a hypoxic workstation (BioSpherix Ltd.; Redfield, NY, USA) under 8% oxygen and 5% CO_2_ in a humidified atmosphere at 37°C for 24 h.

### Preparation of platelet-derived extracellular vesicles

Platelets from healthy pregnant (n = 5) and non-pregnant (n = 3) individuals were isolated as previously described (Forstner *et al.*, 2023) and subsequently activated with 1 IU/ml thrombin (Sigma-Aldrich) in order to release platelet-derived factors and platelet-derived fragments, like extracellular vesicles.

After thrombin activation, platelets were pelleted at 1962 g for 15 min and the supernatant was further centrifuged at 100 000 g (Beckmann Ultracentrifuge) for 75 min. The supernatant, containing the soluble factors, henceforth referred to as PR soluble factors (SF), was collected. The pellet was then washed in PBS before undergoing another centrifugation step at 100 000 g for 75 min. Afterwards, the supernatant was removed and the pellet, containing platelet-derived extracellular vesicles (EVs), was resuspended in 50 µl PBS and stored at −80°C until further analysis. Patient characteristics of the study group used for isolation of platelet-derived EVs are summarized in Supplementary Table S4.

### Characterization of platelet-derived EVs

Co-expression of tetraspanins from enriched platelet-derived EVs was characterized with the ExoView R200+ instrument (NanoView Biosciences, Boston, MA, USA) using the commercially available Leprachaun Exosome Human Plasma Kit (Unchained Labs, Boston, MA, USA). Samples were diluted 1:30,000 (total volume 50 µl) and incubated on tetraspanin chips imprinted with CD81, CD9, CD63, CD41a, and mouse IgG control antibodies for 16 h according to the manufacturer’s protocol. In brief, sample-treated chips were washed, blocked, and stained with fluorescently labeled antibodies (1:500 dilution) against CD9 (CF488A-labeled), CD63 (CF647-labeled), and CD81 (CF555-labeled) for 1 h at RT (all Unchained Labs). Upon incubation, chips were washed, dried, and imaged with the ExoViewR200+ platform. The resulting images were analyzed using the ExoView Analyzer Software (NanoView Biosciences) with EV size thresholds set to 50–200 nm in diameter.

### Nanoparticle tracking analysis

In order to determine concentration and diameter of platelet-derived EVs isolated from healthy pregnant (n = 4) and non-pregnant (n = 3) women, Nanoparticle Tracking Analysis (NanoSight NS3000; Malvern Panalytical Ltd., Malvern, UK) was used according to the manufacturer’s software manual. All samples were diluted with filtered PBS, and thresholds for particle size (diameter) were set to 0 and 1000 nm. Analysis was done using the built-in NanoSight Software (Malvern Panalytical Ltd.).

### qPCR analysis

Total RNA was isolated from BeWo cells and tissue samples using ExtractMe Total RNA Kit (Blirt, Gdansk, Poland) according to the manufacturer’s protocol. Prior to RNA isolation, tissue samples were homogenized with the TissueLyser LT (Qiagen, Hilden, Germany) and Stainless Steel Beads (5 mm, Qiagen) with a subsequent sonification with a Bioruptur^®^ Pico sonication device (Diagenode, Liège, Belgium) for 10 cycles at 4°C. Before performing a Reverse Transcription of 1 µg total RNA using the High-Capacity cDNA Reverse Transcription Kit (Applied Biosystems, Foster City, CA, USA), quality was checked with Nanodrop (ND-1000, Peqlab Biotechnology GmbH; Erlangen, Germany). Gene expression was analyzed by qPCR using Bio-Rad CFX384 Touch Real-Time PCR Detection System (Bio-Rad; Hercules, CA, USA) and SYBR Green qPCR Kit (Biozym, Vienna, Austria). Ct values were analyzed with CFX Manager 3.1 Software (Bio-Rad). *YWHAZ*, *TBP*, and *GAPDH* were used as reference genes (Supplementary Table S5).

### Immunoblots

For immunoblots, 10% Bis-Tris or 3–8% Tris-Acetate gels (NuPAGE^TM^, Invitrogen^TM^, Thermo Fischer Scientific, Waltham, MA, USA), were loaded with 20 µg of clear protein lysate (detailed description is provided in the Supplementary Materials and Methods) of each sample and after gel electrophoresis, proteins were blotted on a 0.45-μm nitrocellulose membrane (Hybond, Amersham Biosciences, GE Healthcare Life Sciences, Little Chalfont, UK). Five microlitres of prestained protein ladder (Thermo Fisher Scientific, Waltham, MA, USA) and 4 µl of Magic Mark^TM^ protein standard (Thermo Fisher Scientific, Waltham, MA, USA) were used as molecular weight markers. After confirming blotting efficiency with Ponceau-S staining (Sigma-Aldrich), membranes were incubated with primary antibodies, summarized in Supplementary Table S6, overnight at 4°C. Following a 2 h incubation step at RT with HRP-conjugated goat anti-mouse and goat anti-rabbit IgG (both 1:5000, Bio-Rad), immunodetection was performed with a chemiluminescent immunodetection kit (WesternBright, Biozym, Vienna, Austria) according to the manufacturer’s instructions. Images were captured using iBright CL 1000 Imaging System (Thermo Fisher Scientific, Waltham, MA, USA), and band densities were quantified with Image Studio Lite 5.2. (LICORbio, Lincoln, NE, USA) Data are presented as a ratio of the target protein band intensity to that of the reference proteins GAPDH and β-actin (Supplementary Table S6).

### Measurement of secreted PAI-1

After treating BeWo cells with human recombinant TGF-β1 (R&D Systems, Inc.) in a final concentration of 20 ng/ml for 24 h, as described above, cell culture media was collected and centrifuged at 491 g for 5 min in order to remove cell debris. Secreted PAI-1 was measured in clear supernatant using the Human PAI1 ELISA Kit (SERPINE1) (Abcam, Cambridge, UK) according to the manufacturer’s protocol. Data were normalized to total protein and analyzed using the Software Curve Expert Professional 2.4.0. (Hyams Development, Madison, AL, USA*).*

### Measurement of TGF-β1

Endogenous TGF-β1 was measured in FBS (Gibco^TM^, Thermo Fisher Scientific), platelet releasate, and platelet lysates, isolated from healthy pregnant women, using the Human TGF-beta 1 Quantikine ELISA (R&D Systems) according to the manufacturer’s protocol. Data were analyzed using the Software Curve Expert Professional 2.4.0. (Hyams Development).

### Immunohistochemistry

FFPE sections from placenta tissue from the first and third trimester were mounted on Superfrost Plus slides (Menzel-Gläser, Thermo Scientific, Waltham, MA, USA) and afterwards deparaffinized. After standard antigen retrieval, immunohistochemistry was performed using the UltraVision Large Volume Detection System HRP Polymer Kit (Thermo Fisher Scientific, Waltham, MA, USA) as previously described (Blaschitz *et al.*, 2015). For immunofluorescence double staining, slides were stained as previously described (Lyssy *et al.*, 2025)., All images were obtained with an SLIDEVIEW^TM^ VS200 Slide Scanner (Evident Europe GmbH, Hamburg, Germany). Detailed protocols for these methods are provided in the Supplementary Materials and Methods and the antibodies used are summarized in Supplementary Table S7.

### Software-based quantification of fibrin

Placental tissue from first and third trimester was either fluorescence double-stained for fibrin and the platelet marker CD42b or immunohistochemical stained for CD42b, as previously described. The antibody used for fibrin detection (see Supplementary Table S7), a mouse monoclonal antibody, has been reported to specifically detect fibrin even in the presence of human fibrinogen and targets specifically an epitope within a heptapeptide from the N-terminal region of the β-chain (Hui *et al.*, 1983). Immunofluorescence images of the whole section were obtained using the SLIDEVIEW^TM^ VS200 Slide Scanner (Evident Europe GmbH) and brightfield images were taken with the Olympus BX63 microscope (Olympus Corporation, Tokyo, Japan). Afterwards, images were subjected to Visiopharm (Version 2021.09, Hørsholm, Denmark) and CellProfiler (Version 3.1.9, Broad Institute, Cambridge, MA, USA) for a software-based quantification of fibrin and adherent platelets. Fibrin area (µm^2^) was normalized to total villi area (µm^2^) and adherent platelets were detected by measuring CD42b positive signals on the villi surface in relation to the total villi surface. A detailed description of the quantification method is provided in the Supplementary Materials and Methods. Patient characteristics of the study group are summarized in Supplementary Tables S1 and S8.

### Statistics

Data were analyzed using GraphPad Prism (Version 10.4.1, GraphPad Software, San Diego, CA, USA) and are presented as means ± SEM. Normality was tested using Shapiro-Wilk and D’Agostino normality test and outliers were identified with Grubbs method (α = 0.05). Significance was tested using repeated-measurement, ordinary one-way ANOVA or *t*-test. One sample *t*-test was used when controls were set as 1. A *P-*value of less than 0.05 was considered statistically significant.

## Results

### Perivillous platelet adherence and fibrin deposition increase over gestation

**Figure 1.**
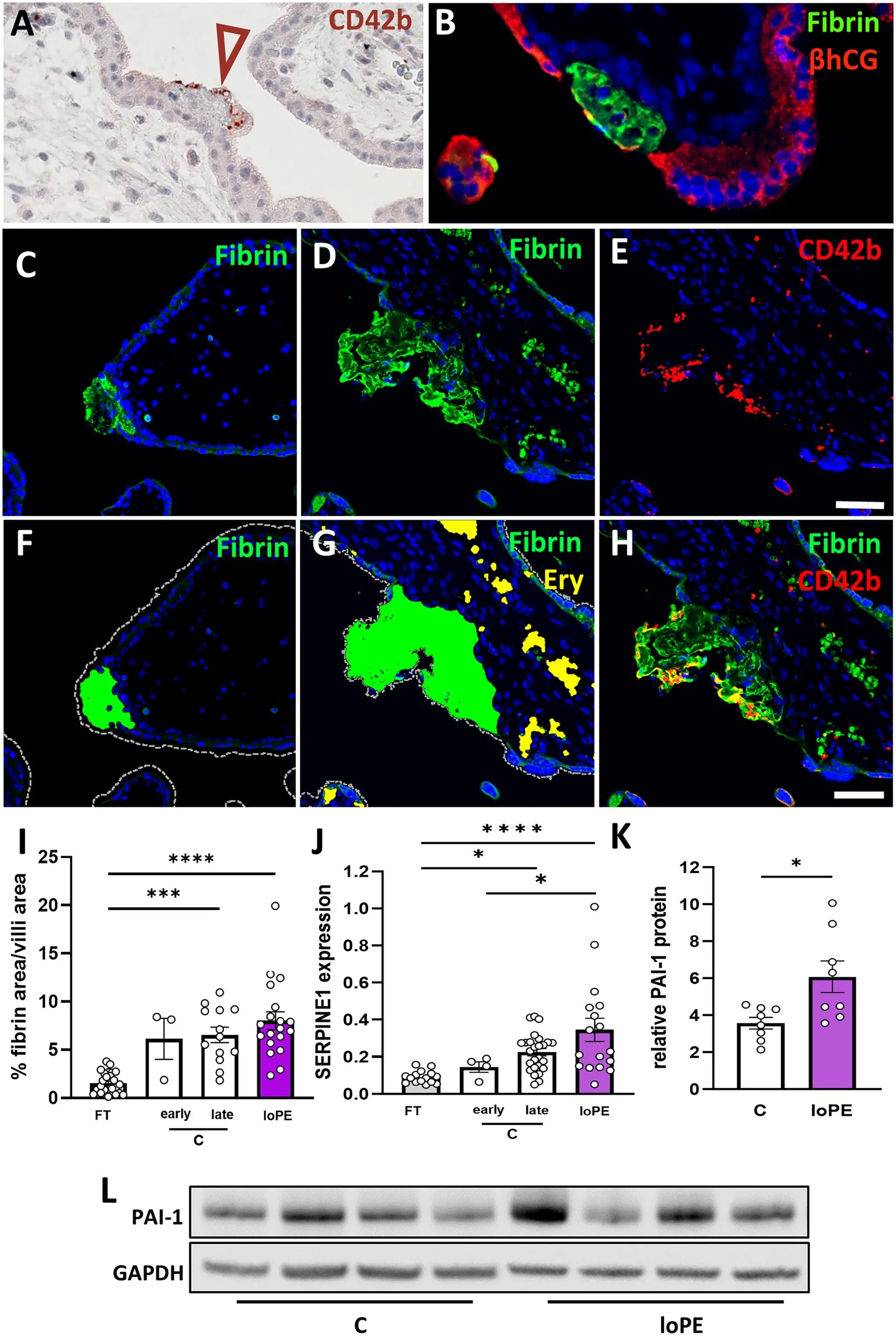
**Placental perivillous fibrin deposition, platelet adhesion, and PAI-1 increase with gestational age.** Human first trimester (FT) placental tissue was subjected to immunohistochemistry for platelet marker CD42b (**A**, Gestational age (GA) 10 + 0). Staining revealed perivillous platelet adhesion (arrowhead). Double immunofluorescence of first trimester placental tissue for fibrin and syncytiotrophoblast (STB) marker human chorionic gonadotropin beta subunit (β-hCG) (**B**, GA 9 + 4) showed a degeneration of the STB and initial perivillous fibrin deposition. A placenta cohort of first trimester tissue (representative image shown in **C**, n = 27), healthy early (n = 3), healthy late (representative image shown in **D**, n = 13) and aged-matched loPE placenta tissue (n = 20) was stained for fibrin (C and D) and subjected to a software-based imaging analysis approach (**F** and **G**). The total area of fibrin (F and G, green area) was normalized to the total villi area (grey lining) and revealed a significant increase of fibrin deposition throughout gestation (**I**). Autofluorescence of erythrocytes in blood vessels were excluded from the analysis (yellow area; G). In term placental tissue (D, **E** and **H**; GA 37 + 0) double immunofluorescence for fibrin and CD42b revealed increased adherence of platelets to more extensive areas of perivillous fibrinoids (H). Placental expression of *SERPINE1* was analyzed in first trimester placental tissue (n = 15), in healthy early (n = 4) and late controls (C) (n = 27) as well as late-onset preeclampsia (LoPE) (n = 17) placentas (**J**). PAI-1 levels were analyzed by immunoblotting in healthy control (n = 8) and preeclamptic (n = 8) placental tissue (**K** and **L**). Data revealed an increase of fibrin, *SERPINE1* and PAI-1 in PE cases. Significance was tested using one-way ANOVA. (I–K). Scale bar represents 100 µm. Data are presented as mean ± SEM. *P ≤ 0.05, ***P ≤ 0.0002, ****P ≤ 0.0001. Ery, Erythrocytes; FT, first trimester.

Initial immunohistochemical staining of human first trimester placenta tissue for the platelet marker CD42b showed platelets on top of homogenous appearing material (Fig. 1A). Subsequent double-staining for fibrin and βhCG, a bona fide marker of the syncytiotrophoblast layer, occasionally revealed degeneration of the syncytiotrophoblast, which was replaced by fibrin deposits, referred to as perivillous fibrinoids (Fig. 1B). Double-staining of term placenta tissue for fibrin and CD42b, verified the adherence of maternal platelets on fibrin-type fibrinoid deposits on the villous surface (Fig. 1C–E).

Since deposition of fibrinoid at the human utero-placental unit is described as a largely normal process (Kaufmann *et al.*, 1996), but has also been positively correlated with pregnancy complications (Lampi *et al.*, 2022), we next investigated the deposition of fibrin throughout gestation and furthermore compared healthy with PE placental tissue. Software-based analysis of healthy placental villi identified a significant increase in fibrin deposits towards term compared to first trimester (Fig. 1F + G), and even a higher increase in age-matched PE samples (Fig. 1H), which also showed a marked rise in adherent platelets (Supplementary Fig. S1). These findings were in agreement with a significant increase in *SERPINE1* expression throughout gestation (Fig. 1I). *SERPINE1* as well as PAI-1 levels, were also significantly higher in placental villi from patients suffering from PE compared to gestational aged-matched controls (Fig. 1I–K, Supplementary Fig. S2). Specificity of the PAI-1 antibody was confirmed by immunoblotting of recombinant PAI-1, revealing a single band at approx. 40 kDa. Notably, PAI-1 in placental tissue lysates was detected at a slightly higher molecular weight (Supplementary Figs S3 and S4). This finding is consistent with the literature and does exclude detection of PAI-1 complexes, such as tPA–PAI-1, which are typically detected at approximately 100 kDa (Brogren *et al.*, 2011). Staining of serial sections of third trimester placental tissue indicates that PAI-1 is predominantly localized to the syncytiotrophoblast, with only minimal association with adhering platelets (Supplementary Fig. S5). While this pattern is consistent with the syncytiotrophoblast being a major contributor to placental PAI-1, it should be noted that PAI-1 can be internalized by cells, as previously shown for HA-tagged PAI-1 in COS7 cells (Garcia *et al.*, 2020), raising the possibility that uptake of PAI-1 from the maternal circulation could in principle contribute to the observed signal.

### Placental PAI-1 expression is upregulated in response to platelet-released factors

**Figure 2.**
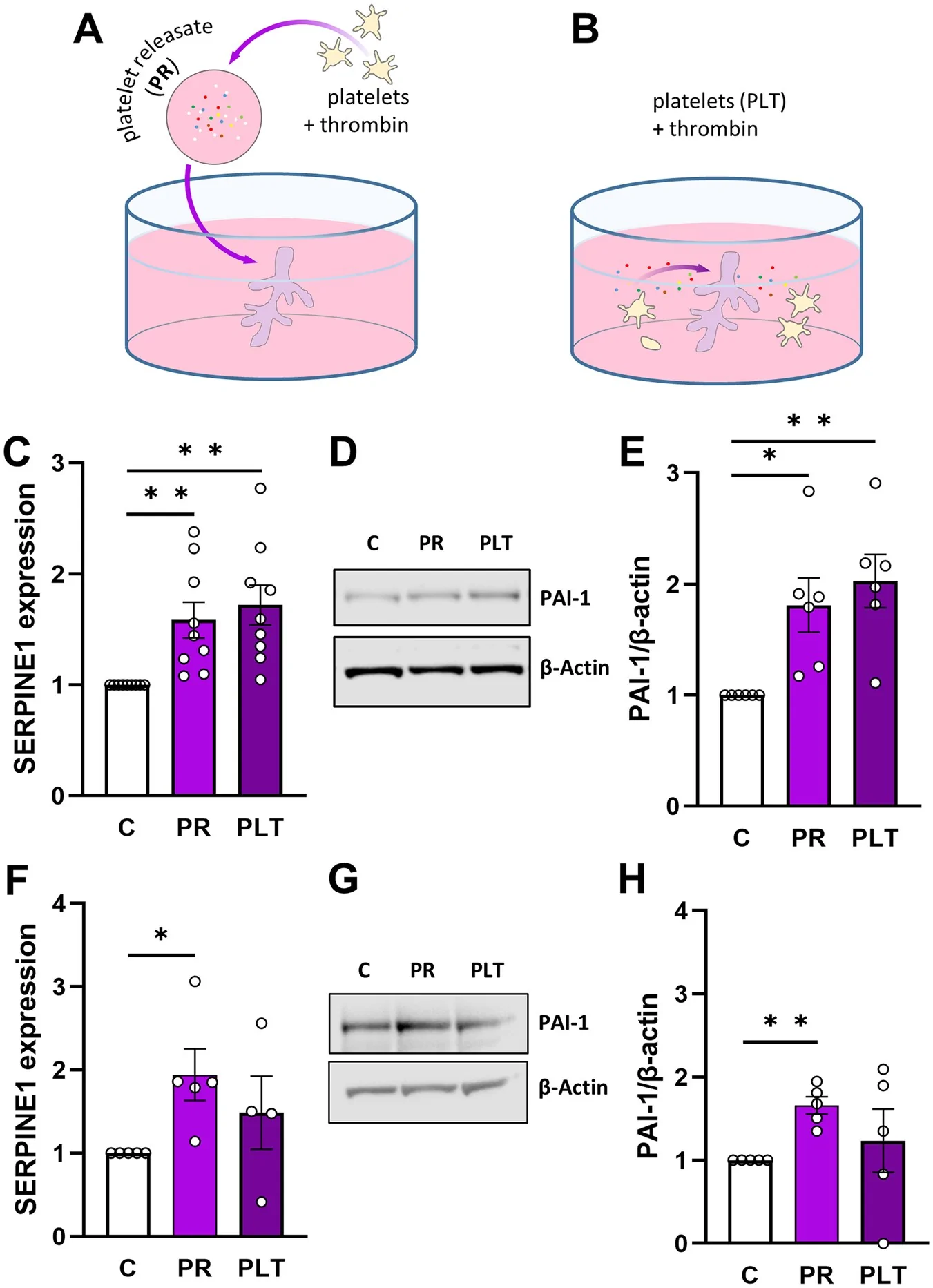
**Platelet-derived factors induce PAI-1 production in human first trimester and term placental tissue.** Human placental villous explant cultures were either incubated for 24 h with platelet releasate (PR) fractions (**A**), or with platelets (PLT) that were directly activated with thrombin in co-culture with placental explants (**B**). Placental explants from the first trimester of pregnancy (n = 9) showed an upregulation of *SERPINE1* (**C**) and PAI-1 protein (**D + E**) (n = 6) in response to both platelet releasate (n = 32) and platelets (n=9) compared to medium control (C). Term placental explants (n = 5) (**F–H**) showed significantly increased *SERPINE1* expression (F) and PAI-1 protein levels (G + H) after incubation with platelet releasate (n = 18), while treatment with washed platelets (n = 5) followed the same trend, though not statistically significant. Significance was tested using a paired *t*-test (C–H). Data are presented as mean ± SEM. *P ≤ 0.05, **P ≤ 0.01.

Next, we aimed to determine the effect of platelet activation on the surface of the syncytiotrophoblast on placental PAI-1 expression. In order to address this study question, two strategies were employed, including incubation of placental explants either with platelet releasate fractions (Fig. 2A) or washed platelets from pregnant women that were activated with thrombin upon co-culture (Fig. 2B). First trimester placenta tissue showed a significant increase in *SERPINE1* expression (Fig. 2C), and its encoded protein PAI-1 (Fig. 2D + E, Supplementary Fig. S6) in response to both PR and direct co-culture with activated platelets. The same trend, although only significant after incubation with platelet releasates, was determined in term placental explants on gene expression as well as on protein levels (Fig. 2F–H, Supplementary Fig. S6). Thrombin alone had no effect on *SERPINE1* expression (data not shown).

### Platelet-derived soluble factors, but not their membrane fractions, are responsible for PAI-1 upregulation

**Figure 3.**
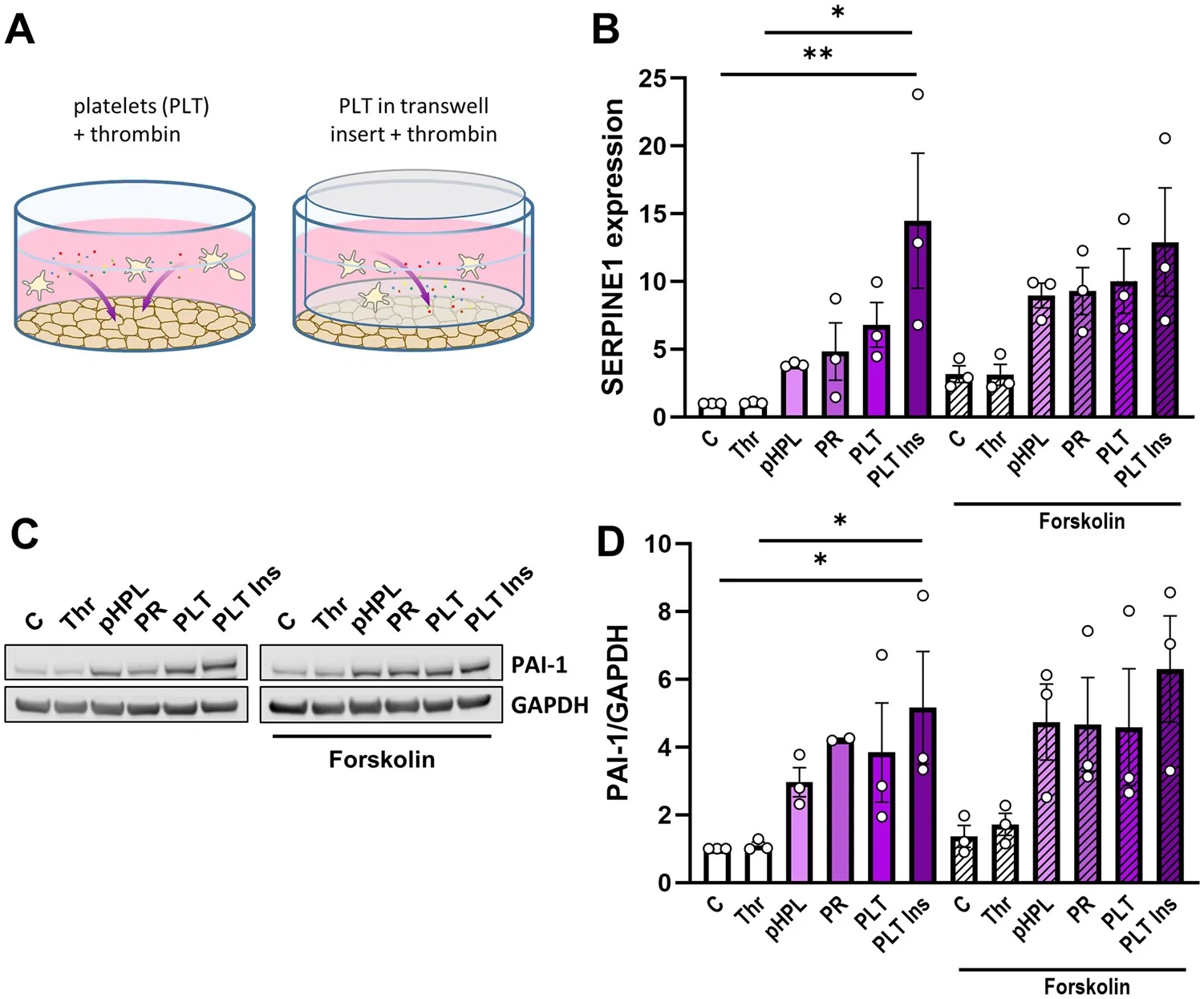
**Platelet-derived soluble factors, but not their membrane fractions, are responsible for trophoblastic PAI-1 upregulation.** BeWo cells were incubated with thrombin-activated platelets either directly in co-culture or indirectly using a transwell system (**A**). Before treatment with platelet products, BeWo cells were incubated with either forskolin (20 µM) to undergo differentiation or solvent control (0.1% v/v DMSO) for 48 h. Pooled human platelet lysate (pHPL) (n = 40), platelet releasate (PR) (n = 7), and thrombin-activated platelets (PLT) (n = 3, mean platelet count 213.33 ± 18.58 × 10^3^ PLT/µl) from healthy pregnant women either directly on cells or in transwell inserts (ins) induced upregulation of *SERPINE1* (**B**) and PAI-1 protein (**C + D**) in both undifferentiated and differentiated BeWo cells after 24 h incubation. Thrombin (Thr) (1 IU/ml) alone had no effect compared to control. Significance was tested using one-way ANOVA method (B + D). Data are presented as mean ± SEM from three independent experiments. *P ≤ 0.05, **P ≤ 0.01. C, control.

**Figure 4.**
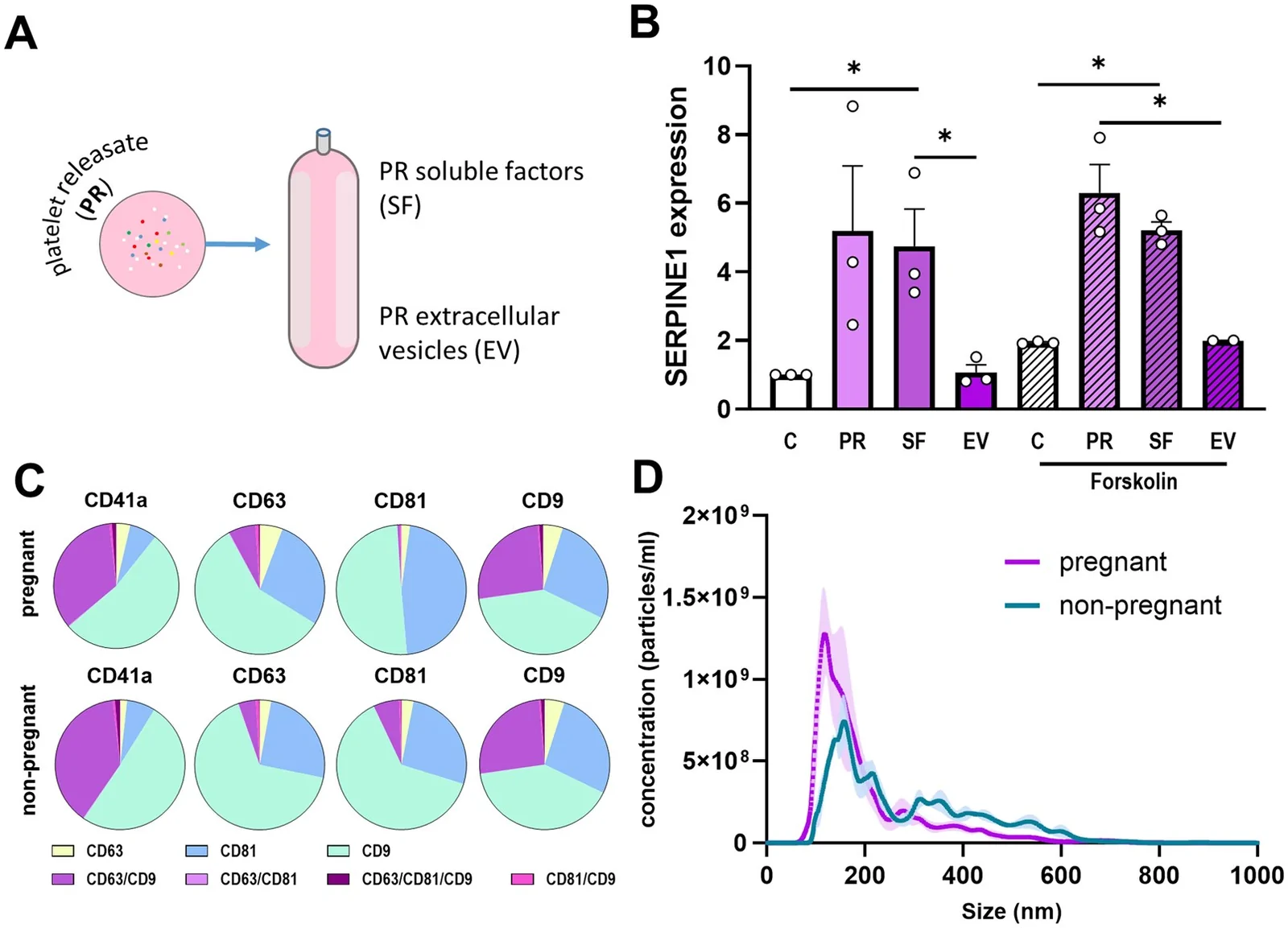
**Platelet-derived extracellular vesicles do not induce trophoblastic SERPINE1 expression.** Platelet releasates (PR) were subjected to ultracentrifugation (**A**) to obtain a fraction of platelet-derived soluble factors (SF) or platelet-derived extracellular vesicles (EV). BeWo cells were incubated with either forskolin (20 µM) to undergo differentiation or solvent control (0.1% v/v DMSO) for 48 h before treatment with either platelet releasate (n = 10), platelet-derived soluble factors or platelet-derived extracellular vesicles for 24 h (**B**). Platelet releasate and soluble factors induced *SERPINE1* upregulation in both undifferentiated and differentiated BeWo cells, whereas platelet-derived EV had no effect compared to controls (B). The co-localization of tetraspanins from enriched platelet-derived extracellular vesicle fractions from pregnant (n = 4) and non-pregnant (n = 3) donors was characterized with the ExoView R200+ instrument. The extracellular vesicles-specific tetraspanins CD63, CD81 and CD9, as well as CD41b, as a platelet marker, were used (**C**). Size distribution and concentration of platelet-derived extracellular vesicle fractions prepared from the same patients’ platelets were determined by Nanoparticle Tracking Analysis (**D**). Data are presented as mean ± SEM from three (B) independent experiments. *P ≤ 0.05.

In order to elucidate whether direct contact of platelets was required for the observed induction of placental PAI-1 production, we next incubated the trophoblast cell line BeWo either with platelets directly added to the cell layer or indirectly within a transwell insert (Fig. 3A). Before, platelets or their products (i.e. pooled lysate or platelet releasates) were added, BeWo cells either underwent forskolin-stimulated differentiation into multinucleated syncytia or remained in an undifferentiated state treated with solvent control. Notably, preincubation with forskolin increased *SERPINE1* expression when compared to undifferentiated controls, regardless of the presence of platelets or their products (Fig. 3B), suggesting good agreement with its predominant expression in the syncytiotrophoblast *in situ*. Additional incubation in the presence of pHPL, PR, or thrombin-activated platelets further increased PAI-1 expression on a transcriptional (Fig. 3B) and protein level (Fig. 3C + D, Supplementary Fig. S7). Amongst treatments, thrombin-activated platelets applied in transwell inserts showed the most pronounced effect, suggesting that neither direct contact of platelets nor their membrane fraction was required for trophoblastic PAI-1 induction. The assumption that only platelet-derived soluble factors were responsible for our observations was further supported by subsequent treatment of BeWo cells with either soluble- or extracellular vesicle preparations obtained from sequential centrifugation of PR (Fig. 4A + B). Incubation of cells with platelet-derived extracellular vesicles did not increase *SERPINE1* expression, whereas PR and PR supernatant, which contained the platelet-derived soluble factors (SF), induced a significant increase in *SERPINE1* expression (Fig. 4B). These findings led to the assumption that platelet-derived soluble factors are responsible for a *SERPINE1* response and not their membrane fractions or direct cell–cell contact. Characterization via co-expression analysis of tetraspanins (CD63, CD9 and CD81) confirmed that the enriched extracellular vesicles are of platelet origin (Fig. 4C and Supplementary Fig. S8).

Notably, nanoparticle tracking analysis suggested that platelet-derived EVs from pregnant women tended to be smaller (D50 156 nm, mode 117 nm) than those from non-pregnant women (D50 243 nm, mode 156 nm), which may reflect pregnancy-related changes in EV biogenesis (Fig. 4D, Supplementary Fig. S8A).

### Platelet-derived TGF-β induces trophoblastic PAI-1 independently of TGF-β receptor 3

**Figure 5.**
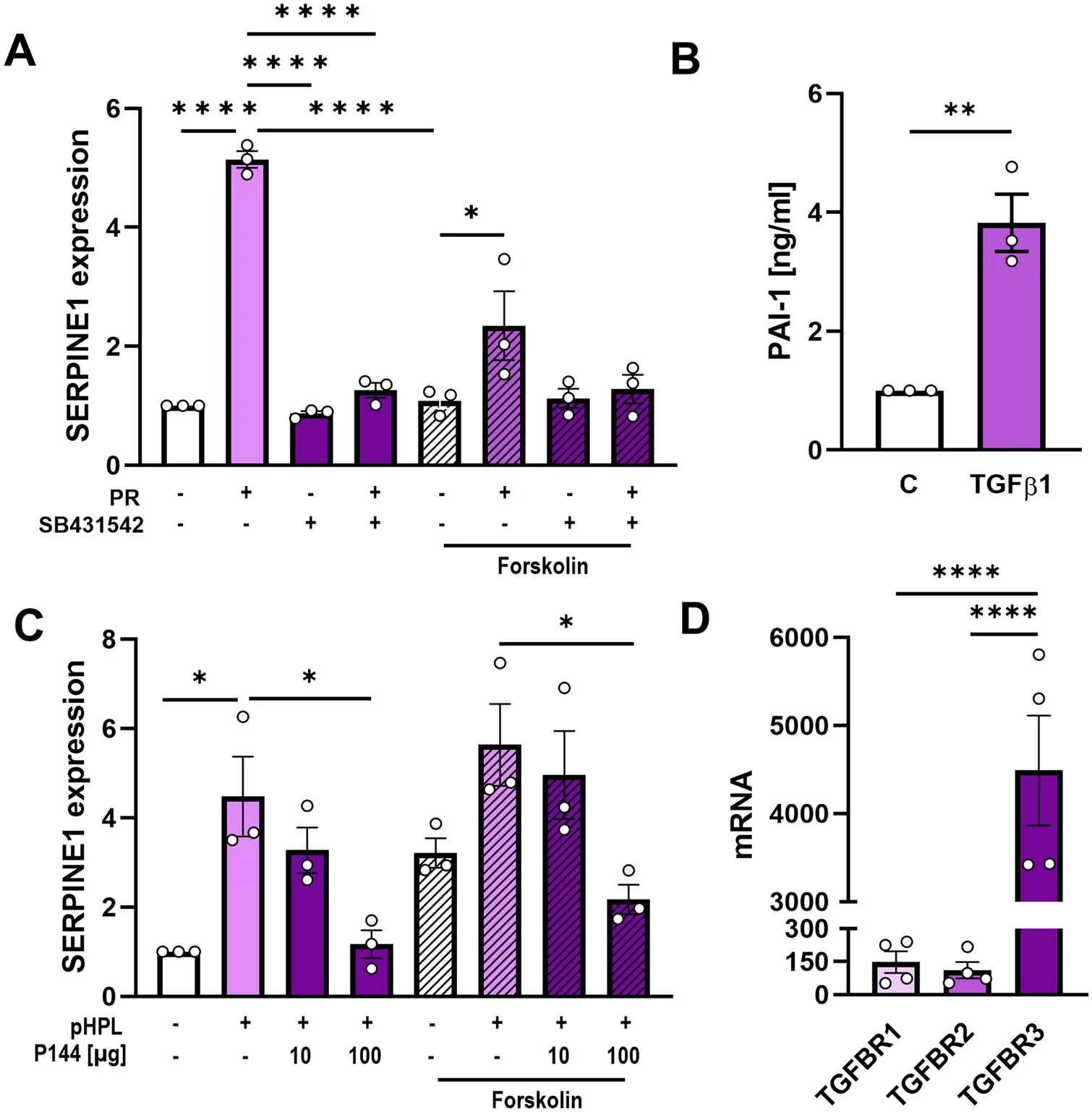
**Platelet-derived TGF-β-signaling induces trophoblastic PAI-1.** BeWo cells were incubated with either solvent control (0.1% v/v DMSO) or forskolin (20 µM) to undergo differentiation for 48 h. Thereafter, cells were pre-incubated with a TGF-β type 1 receptor inhibitor, SB431542 (10 µM) for 2 h, followed by further incubation with or without platelet releasate (PR) (n = 10) in the presence or absence of the inhibitor for 24 h. Presence of SB431542 blocked platelet releasate-induced upregulation of *SERPINE1* to control levels (**A**). Incubation of BeWo cells with recombinant human TGF-β (20 ng/ml) showed an increased secretion of PAI-1, measured via ELISA, after 24 h (**B**). Differentiated BeWo cells and controls were incubated with pooled human platelet lysate (pHPL) (n = 40) in the absence or presence of the peptide P144 (10 and 100 µg/ml), an inhibitor of TGF-β signaling, which blocked the platelet lysate-induced *SERPINE1* expression to control levels after 24 h (**C**). Reevaluation of previous microarray data (Gauster *et al*., 2018) showed abundant TGFBR3 expression in BeWo cells (**D**). Data are presented as mean ± SEM from three (A–C) and four (D) independent experiments. *P ≤ 0.05, **P ≤ 0.01, ****P ≤ 0.0001, TGFBR1, TGF-β type 1 receptor; TGFBR2, TGF-β type 2 receptor; TGFBR3, TGF-β type 3 receptor.

Since platelet α-granules are rich in TGF-β, and this growth factor is a well-known regulator of PAI-1, we next tested whether TGF-β within platelet releasates was the key driver of trophoblastic PAI-1 induction in response to platelet-derived soluble factors. SB431542, an inhibitor of TGF-β receptor signaling, strongly inhibited the upregulation of *SERPINE1* by PR in both undifferentiated and forskolin-stimulated BeWo cells that were incubated in the presence of PR (Fig. 5A). In agreement with this observation, recombinant human TGF-β1 provoked PAI-1 secretion in BeWo cells (Fig. 5B), and overall suggested a regulation through the TGF-β receptor signaling axis. TGF-β1 concentrations of 6.8 ng/ml were measured in pure FBS (100%), whereas PR and platelet lysates contained 19.4 ng/ml and 480.1 ng/ml, respectively. Considering that FBS was used at 10% supplementation, the contribution of endogenous TGF-β1 in the culture medium is likely negligible. However, experiments conducted in the presence or absence of 10% FBS showed that *SERPINE1* induction in response to platelet-derived factors was more pronounced in the presence of FBS (Supplementary Figs S9 and S10).

Considering that SB431542 is also reported to inhibit activin and bone morphogenetic proteins (BMP) signaling, we next applied P144 (Disetertide), a TGF-β inhibitor specifically designed to block the interaction of TGF-β with its receptor (Gallo-Oller *et al.*, 2016). P144 administration significantly reversed pHPL-mediated *SERPINE1* induction to control levels, irrespective of whether BeWo cells underwent differentiation or not (Fig. 5C). P144 is a peptide (TSLDASIIWAMMQN) derived from the sequence of the extracellular region of TGF-β receptor 3 (Cruz-Morande *et al.*, 2022), which is why we next hypothesized whether TGF-β receptor 3 was involved in trophoblastic PAI-1 regulation.

**Figure 6.**
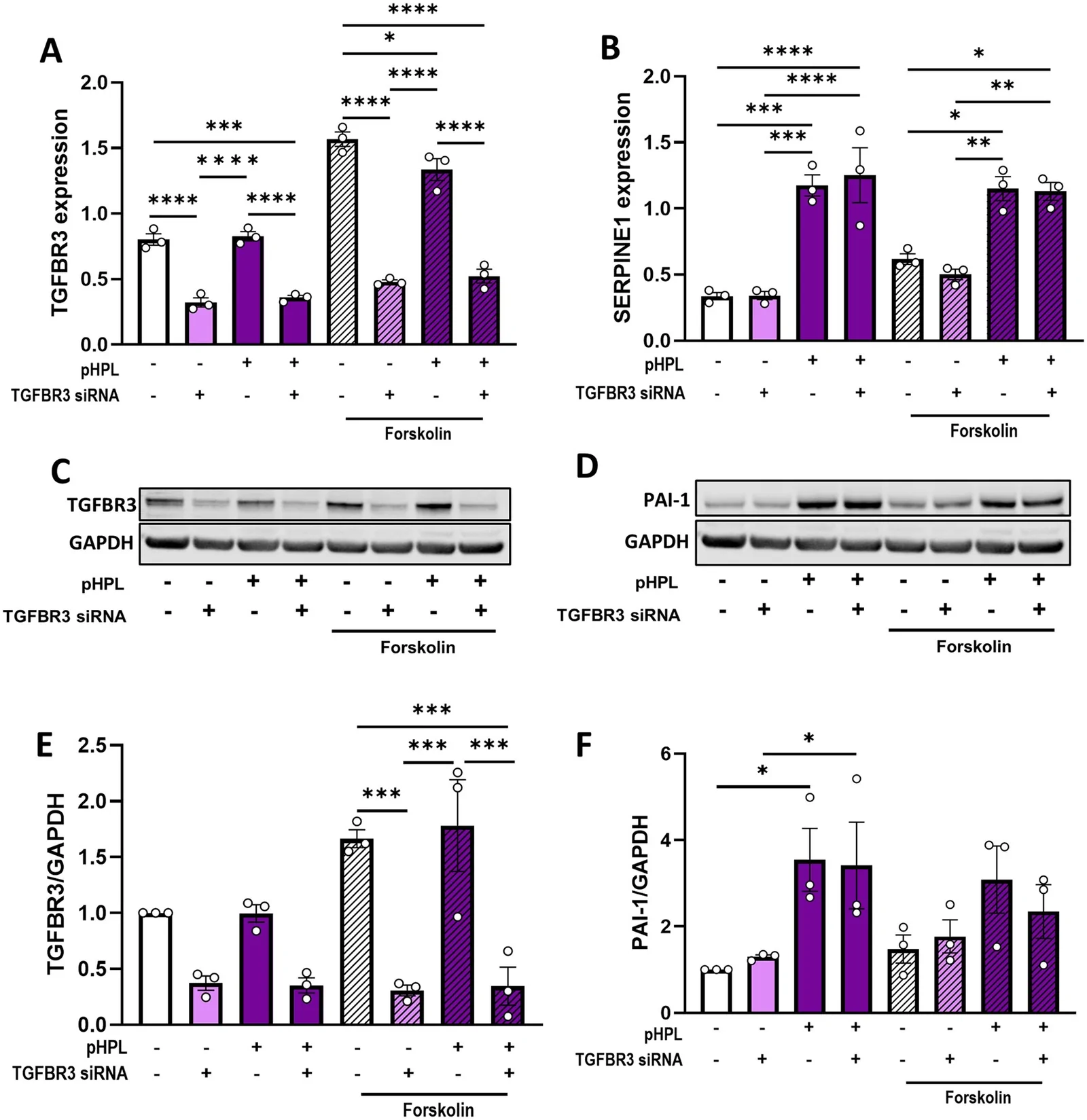
**Induction of trophoblastic PAI-1 by platelet-derived factors is independent of the TGF-β type 3 receptor.** Expression of TGF-β type 3 receptor was silenced in BeWo cells for 24h, followed by incubation with forskolin (20 µM) or solvent control (0.1% v/v DMSO), in the presence or absence of platelet lysate (pHPL) for an additional 24h. Efficiency of TGF-β type 3 receptor silencing was confirmed on mRNA (**A**) and protein level (**C** + **E**). Silencing of TGF-β type 3 receptor had no effect on platelet lysate-induced upregulation of *SERPINE1* (**B**) and PAI-1 levels (**D** + **F**), compared to controls. Data are presented as mean ± SEM from three (**A-F**) independent experiments. *P ≤ 0.05, **P ≤ 0.01, ***P ≤ 0.001, ****P ≤ 0.0001, TGFBR3, TGF-β type 3 receptor.

Initial screening of our previous microarray data (NCBI GEO Accession #: GSE98523; Gauster *et al.*, 2018) confirmed abundant expression of *TGFBR3* that far exceeded that of *TGFBR1* and *TGFBR2* (Fig. 5D). Notably, forskolin-induced differentiation of BeWo cells was accompanied by an upregulation of the receptor, both on mRNA (Fig. 6A) and on protein level (Fig. 6C + E, Supplementary Fig. S11). Using siRNA approach, *TGFBR3* expression was efficiently silenced by more than 50% in both differentiated and control cells at mRNA and protein levels (Fig. 6A, C and E). However, silencing of *TGFBR3* expression had no effect on pHPL-induced upregulation (Fig. 6B) and synthesis of PAI-1 (Fig. 6D + F, Supplementary Fig. S11), suggesting that the receptor was not directly involved in this process. However, silencing of TGFBR1 revealed a substantial decrease in *SERPINE1* expression and PAI-1 levels upon incubation with either recombinant human TGF-β1 or PR, indicating an integral mechanistic role of this receptor in the regulation of placental PAI-1 (Supplementary Figs S12 and S13).

## Discussion

Here, we show that platelet-derived TGF-β released upon platelet activation drives placental PAI-1 production via signaling through TGFBR1, while this effect does not require the co-receptor TGFBR3. This assumption is based on the fact that blocking TGF-β signaling by either SB431542 or P144 impaired trophoblastic PAI-1 in response to platelet-derived soluble factors, but not the platelet-derived EV fraction. Moreover, our data underpin the concept that TGFBR3 can act as a dual modulator. While silencing of the receptor had no direct effect, P144, a small synthetic peptide derived from the extracellular region of the TGFBR3 receptor, significantly impaired PAI-1 induction. This suggests that TGF-β is sequestered by an excess of P144 in our experimental setup, but more importantly tempts us to speculate about an indirect fine-tuning role of the soluble variant of TGFBR3, which is reported to be released into circulation by ectodomain shedding. Extracellular domain modifications, such as glycosaminoglycans, may affect ectodomain shedding (Choi *et al.*, 2024), which could be the underlying reason for increased maternal circulating levels of soluble TGFBR3 (soluble betaglycan) that are reported for severe PE (Wang *et al.*, 2017; Xu *et al.*, 2017), while its expression is not significantly altered in PE placental tissue (Casagrandi *et al.*, 2003). As to why trophoblastic *SERPINE1* upregulation was most pronounced when platelets were applied and activated in a transwell system remains speculative. However, a potential explanation may relate to differences in the availability, kinetics, and duration of exposure to platelet-derived soluble factors. In the transwell, physical separation may allow factors to diffuse more uniformly and persist in the medium, providing trophoblasts with a more sustained exposure.

Maternal platelets are suggested as the main source of TGF-β in the circulation, as TGF-β plasma levels were reported to correlate with both platelet concentration and aggregation ability (Peraçoli *et al.*, 2008). In patients with PE, the maternal mean platelet volume (MPV) and the immature platelet fraction (IPF) are increased, whereas total platelet counts are reduced (Woldeamanuel *et al.*, 2023), which may be explained by increased platelet consumption in peripheral tissues, including the placenta. The increased consumption may start early in pregnancy, since the MPV and platelet distribution width (PDW) have been shown to be increased at 14–18 weeks of gestation in women who later on developed early onset PE (Udeh *et al.*, 2024). The consequence of increased consumption is that the bone marrow is forced to produce fresh young platelets, eventually leading to exhaustion of the system. Recent transmission electron microscopy suggests an “exhausted” state of platelets in PE patients, showing signs of previous activation, with increased filopodial protrusions and remarkable ultra-structural remodeling of the open canalicular system (Agbani *et al.*, 2023). Thus, increased platelet activation and consumption may lead to increased circulating TGF-β levels in PE, as reported by the vast majority of previous studies (Horvat Mercnik *et al.*, 2024).

Increased circulating TGF-β may not only be the trigger for increased placental PAI-1 production, but also for connective tissue growth factor (CTGF), which is reported to be elevated in placental tissue and serum of patients with severe PE and fetal growth restriction (Oh *et al.*, 2009). Whether increased placental PAI-1 is one of the causal reasons for the development of PE, or just the consequence thereof, remains a matter of discussion. However, our *in vitro* experiments with peptide P144 tempt us to speculate on therapy options for blocking TGF-β-induced placental PAI-1 production in PE pregnancies. Besides inhibition of TGF-β signaling, another, more specific option could be a direct inhibition of PAI-1. So far, a wide collection of approaches, including small molecules, synthetic peptides, RNA aptamers, monoclonal antibodies, and antibody derivatives, have been developed, but despite their extensive *in vitro* and *in vivo* characterization, no PAI-1 inhibitor is currently approved for therapeutic use in humans (Sillen and Declerck, 2020). The observation that platelet activation drives placental PAI-1 expression may lead one to speculate whether this provides a mechanistic explanation for the clinical efficacy of low-dose aspirin in pregnancies at high risk for the development of PE (Mayer-Pickel *et al.*, 2019). Indeed, aspirin has been described to reduce systemic PAI-1 levels by inhibiting the immediate release of PAI-1 from platelet α-granules (Colwell, 2003). However, although aspirin inhibits COX-1–dependent platelet activation and thereby reduces the release of platelet-stored PAI-1, it does not fully prevent activation through alternative pathways and may therefore not sufficiently suppress TGF-β-mediated induction of PAI-1 expression in trophoblasts. However, it should be mentioned that in addition to TGF-β, also mechanical forces such as shear strain and cyclic stretching, which could alter cell shape by disrupting actin-based and microtubule networks, are suggested to induce PAI-1 (Samarakoon *et al.*, 2010). Thus, increased mechanical stress by velocity jets and elevated wall shear stress on the placental surface, as suggested by an *in silico* dynamic flow model for pregnancies complicated by intrauterine growth restriction (IUGR) and/or PE (Roth *et al.*, 2017), could be another trigger for increased placental PAI-1 production.

Our analysis of platelet-derived EVs revealed a particle distribution around 150 nm, which is within the reported range of 100–250 nm (Moon *et al.*, 2024). While we observed no effect on placental PAI-1 expression, previous studies suggest that microvesicles (100–200 nm) can deliver TGF-β and activate SMAD3 signaling in endothelial progenitor cells (Yan *et al.*, 2020). Although our sample size is small, the smaller EVs observed in pregnancy may reflect the hypercoagulable state, consistent with reports that large EVs originate from the platelet plasma membrane and smaller EVs from internal storage vesicles such as α granules (Dean *et al.*, 2009).

The fact that thrombin has previously been shown to induce the production of PAI-1 in bovine aortic smooth muscle cells (Noda-Heiny *et al.*, 1993), and that PR fractions for our current study were prepared with thrombin, may raise the question as to whether observed effects were mediated by thrombin rather than platelet-released factors. However, in experiments designed to assess the sole effects of thrombin and hirudin, no changes in *SERPINE1* expression were observed compared to controls, thereby arguing against this possibility. Additionally, since hirudin, a specific inhibitor of thrombin, was added shortly after platelet activation in our releasate preparation protocol, we therefore exclude a thrombin-mediated effect. This notion is supported by a previous study, demonstrating that preincubation of thrombin with hirudin prevented the induction of PAI-1 synthesis (Noda-Heiny *et al.*, 1993), and is confirmed by our own experiments with thrombin that was preincubated with hirudin, showing no effects on trophoblastic PAI-1 expression.

We noted that platelet adherence to perivillous fibrinoids can occur even in early gestation, though the underlying mechanisms remain unclear (Pierleoni *et al.*, 2003). Fibrin deposition on villous surfaces is normally observed in ∼7% of term placentas (Nelson *et al.*, 1990), and appeared increased in our PE cases. While extensive perivillous fibrin has been suggested to associate with placental malperfusion and adverse outcomes such as PE and FGR (Spinillo *et al.*, 2019), these observations are exploratory and outside the primary conclusions of this study.

In conclusion, our study has generated novel insights into the regulation of placental PAI-1 by platelet-derived TGF-β. We could show that TGF-β receptor 3 is not involved in TGF-β-mediated PAI-1 induction in trophoblasts and that platelet-derived EVs, generated by thrombin activation, do not trigger trophoblastic PAI-1 production.

## Supplementary Material

gaag040_Supplementary_Data

## Data Availability

The data underlying this article will be shared on reasonable request to the corresponding author.

## Author Biography

**Dr.** **Désirée Forstner:** Désirée Forstner, PhD, is a Senior Lecturer at the Division of Cell Biology, Histology and Embryology at the Medical University of Graz (Austria) and a member of the placenta research team. She completed her doctoral thesis focusing on the role of maternal platelets in trophoblast differentiation. During her doctoral studies, she conducted a research stay at the University Hospital Leipzig (Germany), further expanding her expertise in platelet biology. Her scientific work focuses on the interplay between pregnancy complications and platelet dysregulations, particularly in preeclampsia, with a special emphasis on trophoblast function and differentiation. Dr. Forstner has received scientific awards, authored peer-reviewed publications, and is a member of the European Placenta Group.
