# Supplementary material for: Placental plasminogen activator inhibitor 1 is induced by platelet-derived TGF-β independently of TGF-β receptor 3 and is upregulated in preeclampsia
Source: Mol Hum Reprod. 2026 Jun 29;32(3):gaag040. doi: 10.1093/molehr/gaag040 (PMC13387163; doi:10.1093/molehr/gaag040)
Supplement: gaag040_Supplementary_Data [file gaag040_supplementary_data.pdf]

Placental plasminogen activator inhibitor 1 is induced by platelet-derived TGF-β independently of TGF-β receptor 3 and is upregulated in preeclampsia

Désirée Forstner, Azra Kulovic-Sissawo, Freya Lyssy, Jacqueline Guettler, Djenana Vejzovic, Beate Rinner, Daniel Kummer, Nadja Kupper, Christina Stern, Michael Gruber, Lena Neuper, Christine Daxboeck, Anubhuti Gupta, Shrey Kohli, Berend Isermann, Martin Gauster

Supplementary Figure S1: Software-based quantification of adherent platelets on placental villi

Supplementary Figure S2: Full sized image of western blot shown in Figure 1K

Supplementary Figure S3: Validation of the anti-PAI-1 antibody

Supplementary Figure S4: Full sized image of western blot shown in Supplementary Figure 3A+B

Supplementary Figure S5: Expression of PAI-1 at the apical side of the syncytiotrophoblast

Supplementary Figure S6: Full sized image of western blot shown in Figure 2D+G

Supplementary Figure S7: Full sized image of western blot shown in Figure 3C

Supplementary Figure S8: Characterization of platelet-derived extracellular vesicles

Supplementary Figure S9: PAI-1 induction by platelet-derived factors is largely independent of FBS

Supplementary Figure S10: Full sized image of western blot shown in Supplementary Figure 9C

Supplementary Figure S11: Full sized image of western blot shown in Figure 6C+D

Supplementary Figure S12: Upregulation of PAI-1 in response to TGF-β1 and platelet-derived factors is induced via TGFBR1

Supplementary Figure S13: Full sized image of western blot shown in Supplementary Figure 12C+H

Supplementary Table S1: Patient Characteristics of the study group shown in Figure 1

Supplementary Table S2: Patient Characteristics of the study group shown in Figure 2

Supplementary Table S3: Patient Characteristics of the study group used for the preparation of platelet releasate (PR)

Supplementary Table S4: Patient Characteristics of the study group used for isolation and characterization of platelet-derived EVs

Supplementary Table S5: Forward and reverse primer sequences for gene expression analysis

Supplementary Table S6: Primary and secondary antibodies for western blot

Supplementary Table S7: Primary and secondary antibodies for immunofluorescence and immunohistochemistry

Supplementary Table S8: Patient Characteristics of the study group shown in Supplementary Figure 1

Supplementary Materials and Methods

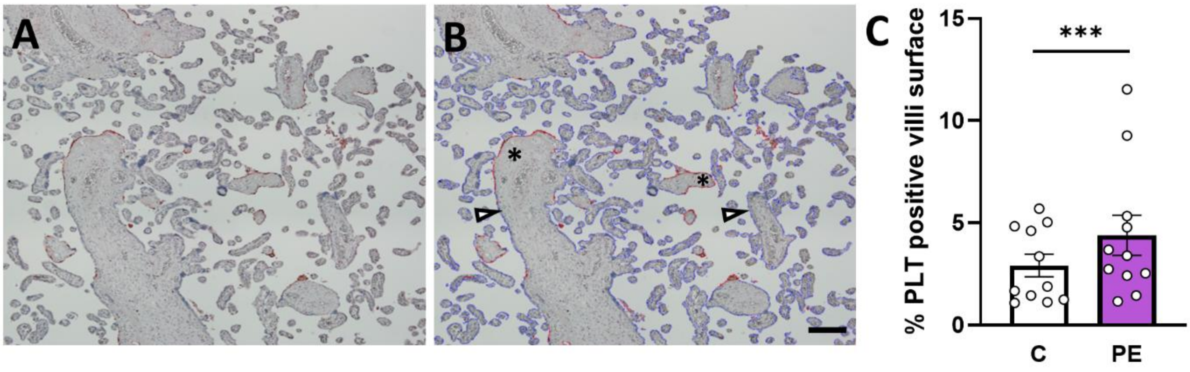

Supplementary Figure S1. *Software-based quantification of adherent platelets on placental villi*

Immunohistochemistry for CD42b (**A**) and a subsequent software-based analysis (Visiopharm 2021.09) of the total villi surface (**B**, blue lining, indicated with arrowhead) and adherent CD42b positive platelet areas (**B**, red lining, indicated with asterisk) of placental villi showed an increase of adherent platelets in preeclamptic tissue (n=11) compared to healthy controls (n=11) (**C**). Scale bar represents 100 µm. ***p ≤ 0.001; C,control; PE, preeclampsia.

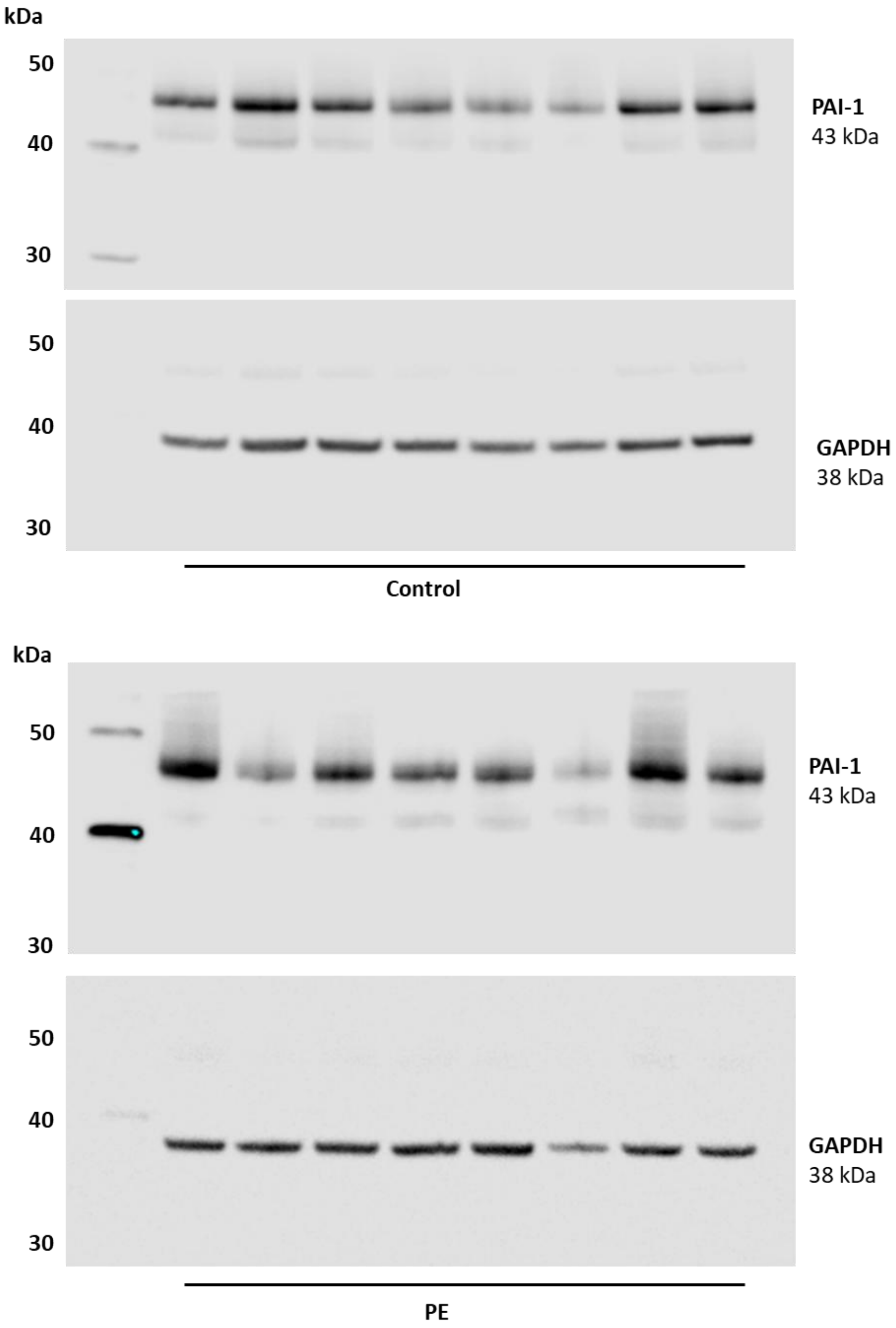

Supplementary Figure S2: Full sized image of western blot shown in Figure 1K; PE, Preeclampsia.

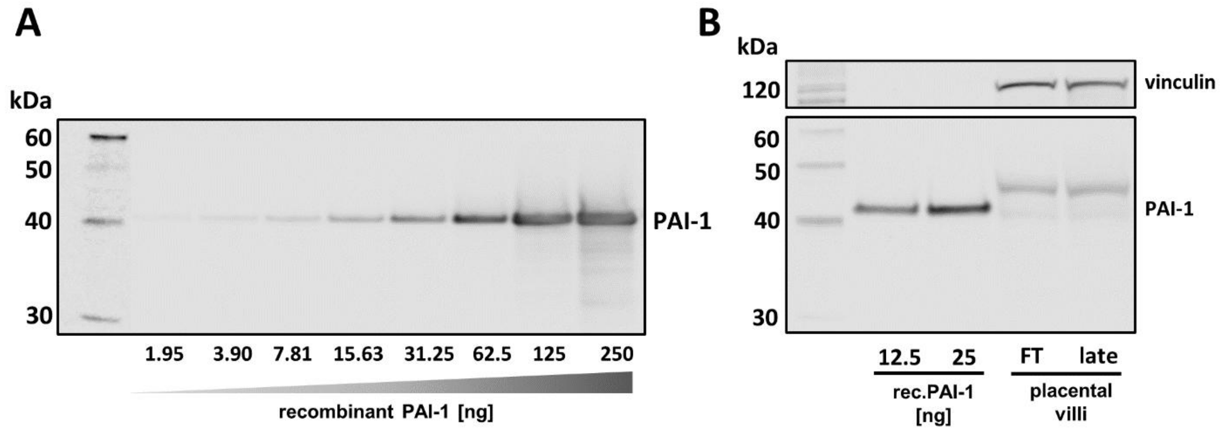

Supplementary Figure S3. *Validation of the anti-PAI-1 antibody*

Serial dilutions of the recombinant PAI-1 protein (Merck, Darmstadt, Germany) in the range of 1.25 to 250 ng were subjected to immunoblotting for PAI-1 and revealed a single band at approximately 40kDa, confirming the specificity of the antibody (**A**). Lysates of placental villi tissue from the first (GA 6+0) and third (GA 40+0) trimester showed a single band at approximately 45 kDa, indicating the expected molecular weight for monomeric PAI-1 (**B**). GA, gestational age; FT, first trimester; late, third trimester.

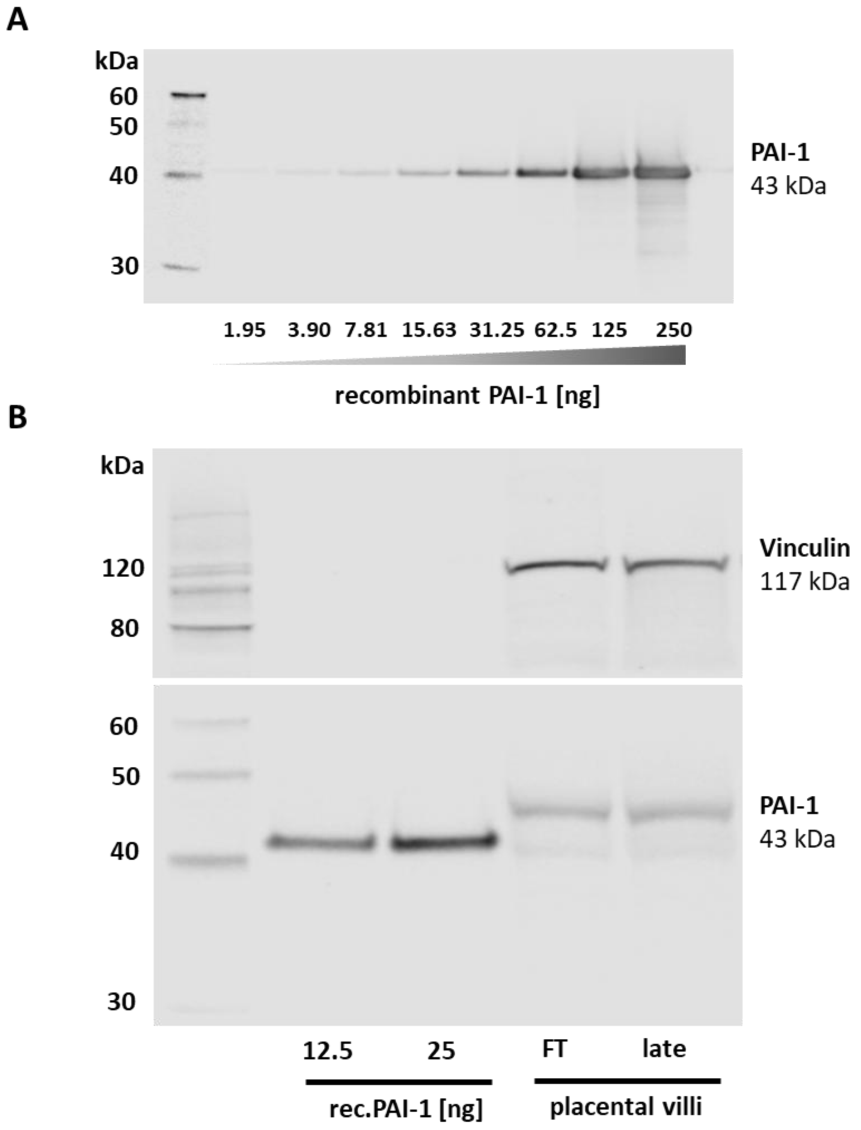

Supplementary Figure S4: Full sized image of western blot shown in Supplementary Figure 3A+B; (**A**) is showing the full sized western blot image of Supplementary Figure 3A and (**B**) is showing the full sized western blot image of Supplementary Figure 3B.

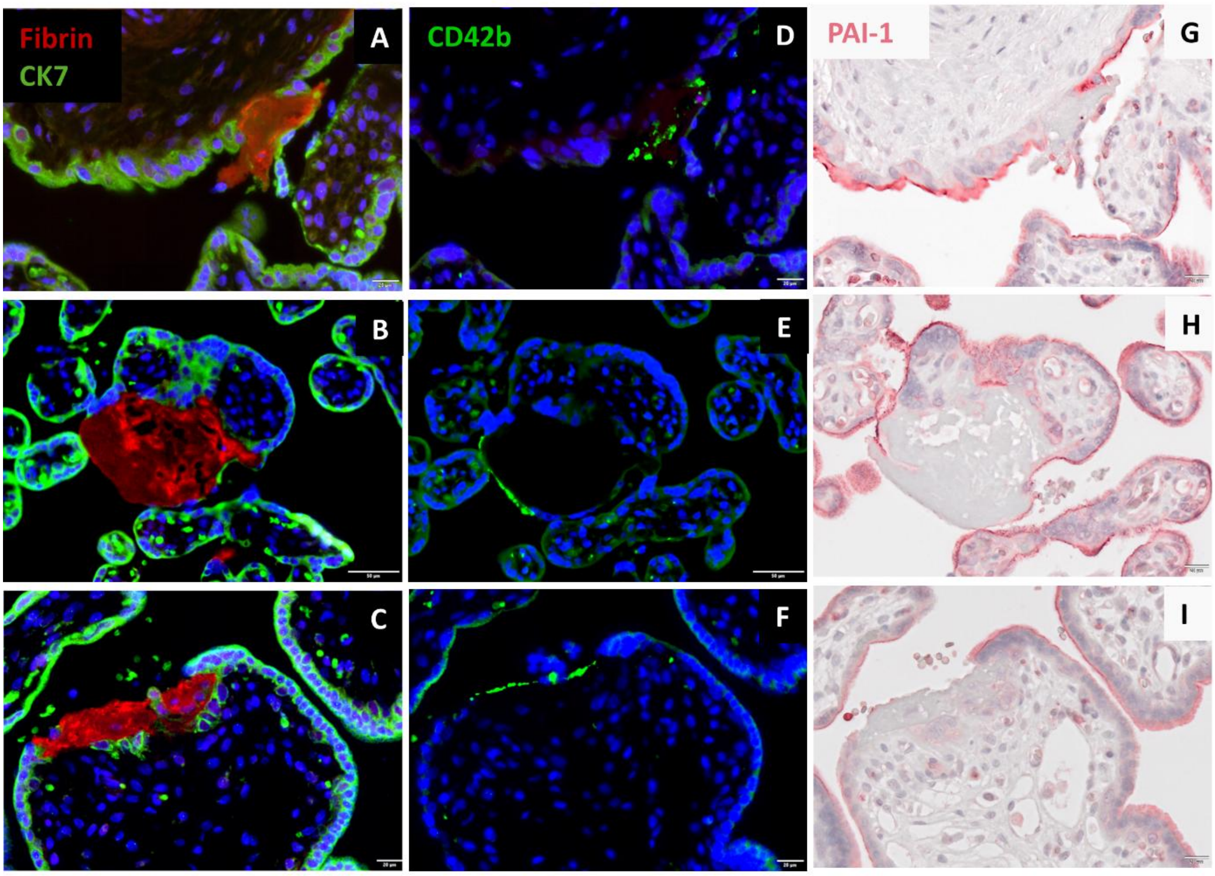

Supplementary Figure S5. *Expression of PAI-1 at the apical side of the syncytiotrophoblast*

Serial sections of third trimester placental tissue were subjected to immunofluorescence double staining for fibrin (red) and the syncytiotrophoblast marker cytokeratin 7 (green) (**A-C**) or IF single staining for CD42b as platelet marker (**D-F**). Immunohistochemistry for PAI-1 (**G-I**) revealed a strong expression of PAI-1 at the apical side of the syncytiotrophoblast, whereas adherent platelets were mainly negative. Scale bar represents 20 µm in A, C, D, F, G and I and 50 µm in B, E and H; CK, Cytokeratin 7.

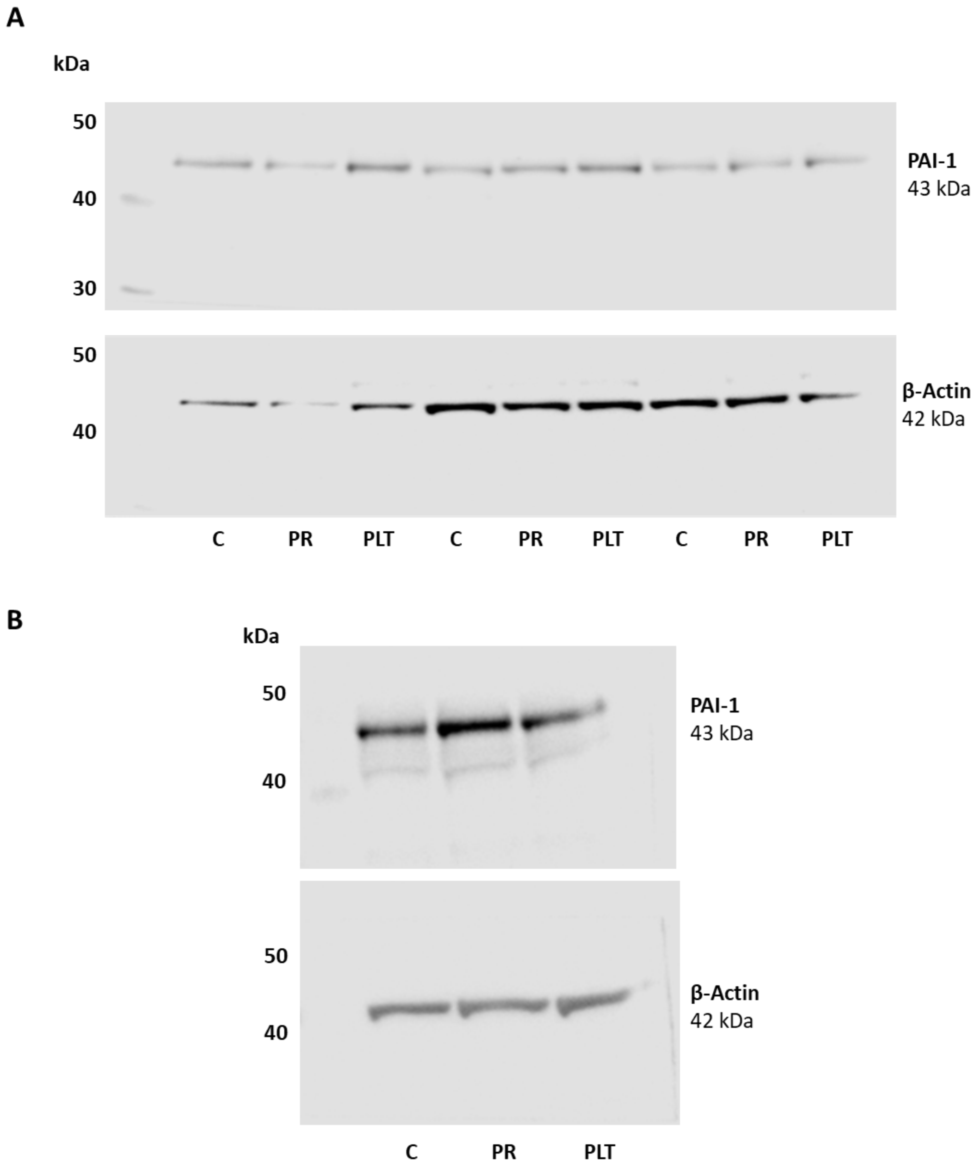

Supplementary Figure S6: Full sized image of western blot shown in Figure 2D+G, (**A**) is showing the full sized western blot image of Figure 2D and (**B**) is showing the full sized western blot image of Figure 2G. C, Control; PR, platelet releasate; PLT, platelets.

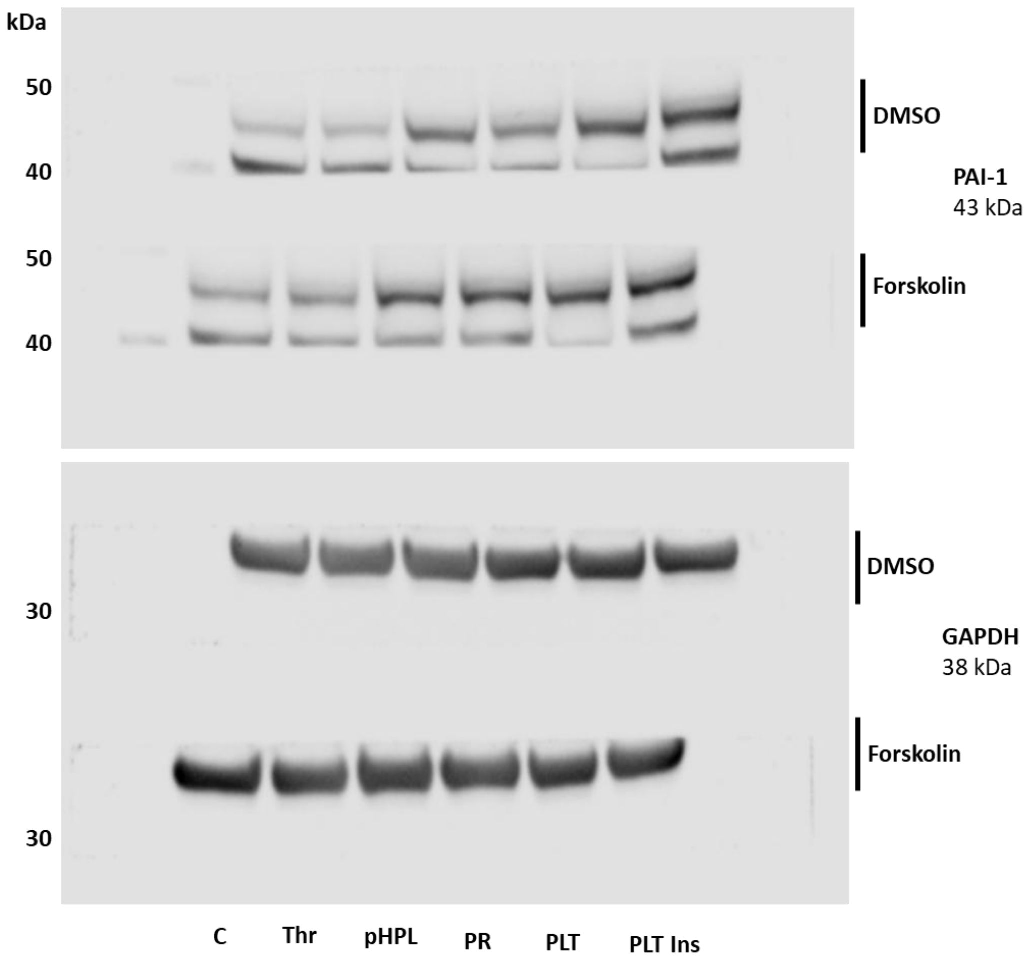

Supplementary Figure S7: Full sized image of the western blot shown in Figure 3C; C, Control; Thr, Thrombin; pHPL, pooled human platelet lysate; PR, platelet releasate; PLT, platelets directly on cells; PLT Ins, platelets in transwell inserts.

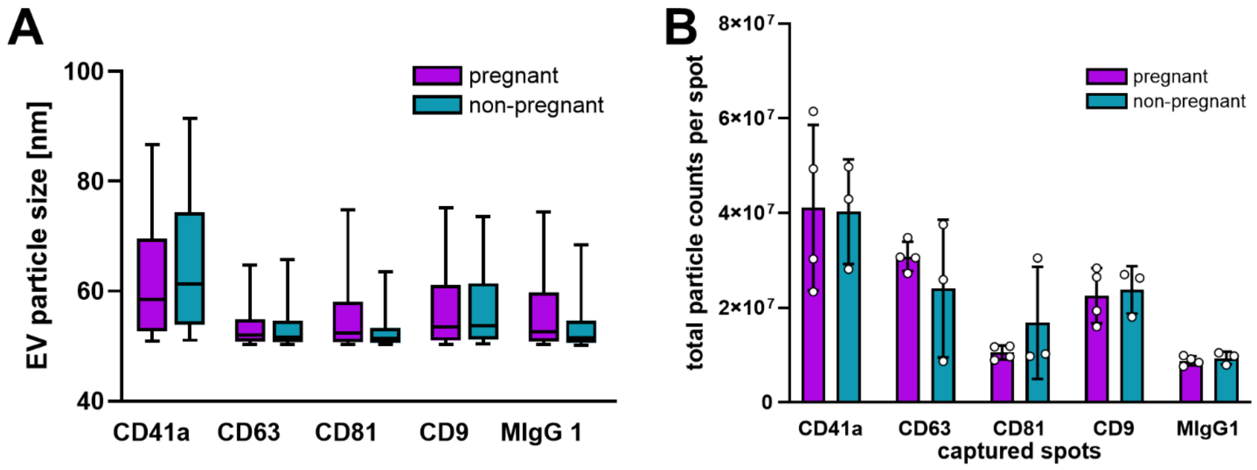

Supplementary Figure S8. *Characterization of platelet-derived extracellular vesicles*

The size distribution of platelet-derived extracellular vesicles fractions from healthy non-pregnant (n=3) and pregnant women (n=4) were identified using ExoView R200+ by plotting reported particle data (Whiskers represent 10th and 90th percentile) (**A**). Total particle counts of the captured spots were analyzed using ExoView R200+ (**B**). MIgG1, Mouse Immunoglobulin G1

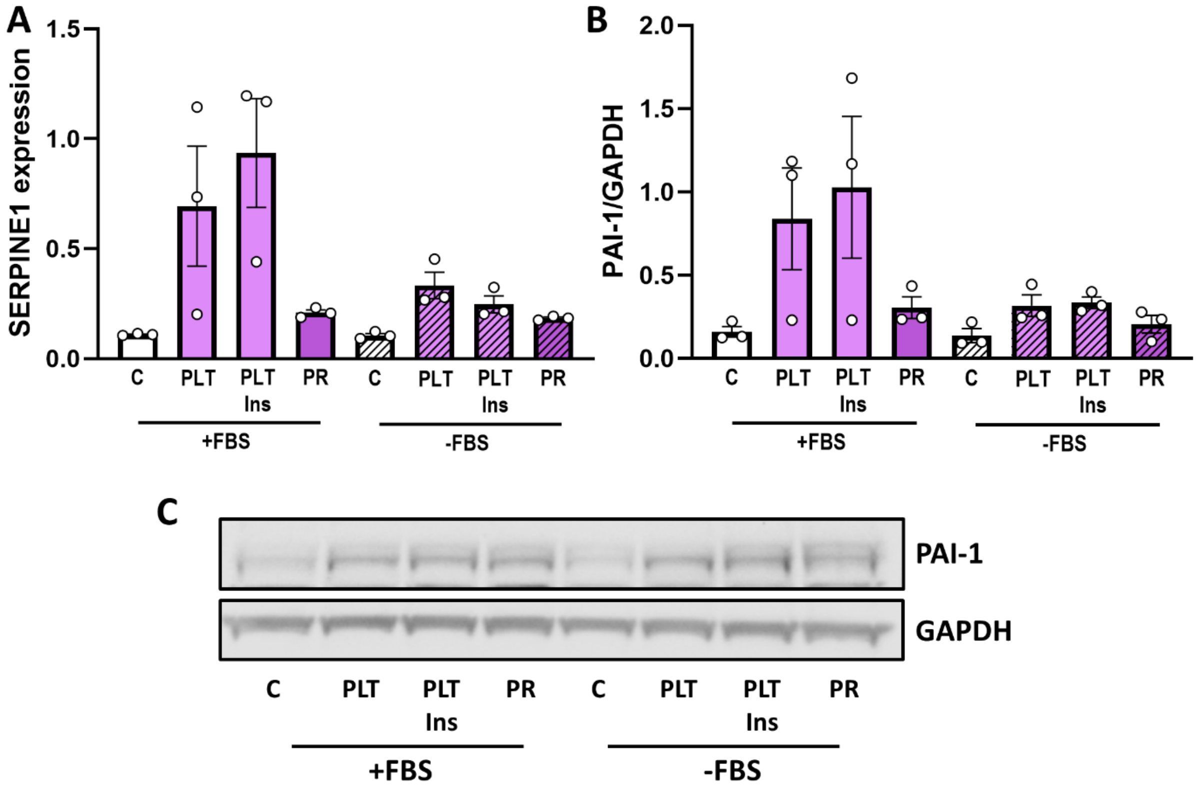

Supplementary Figure S9. *PAI-1 induction by platelet-derived factors is largely independent of FBS*

BeWo cells were seeded in a 24-well plate with a density of 100.000 cells per well overnight before differentiation with forskolin for 48h. After differentiation, cells were incubated with either isolated platelets directly applied to the cell layer or in a transwell insert and platelet releasate in presence or absence of 10% (v/v) FBS (n=3). *SERPINE1* expression (**A**) and PAI-1 (**B** and **C**) in cell lysates was upregulated in response to platelets and platelet-derived factors in both, presence or absence of FBS, although with a higher effect in presence of FBS. Data are represented as mean ± SEM from three independent experiments. Significance was tested using a one-way ANOVA; C, control; PLT, platelets directly on cells; PLT Ins, platelets in transwell insert; PR, platelet releasate; FBS, fetal bovine serum.

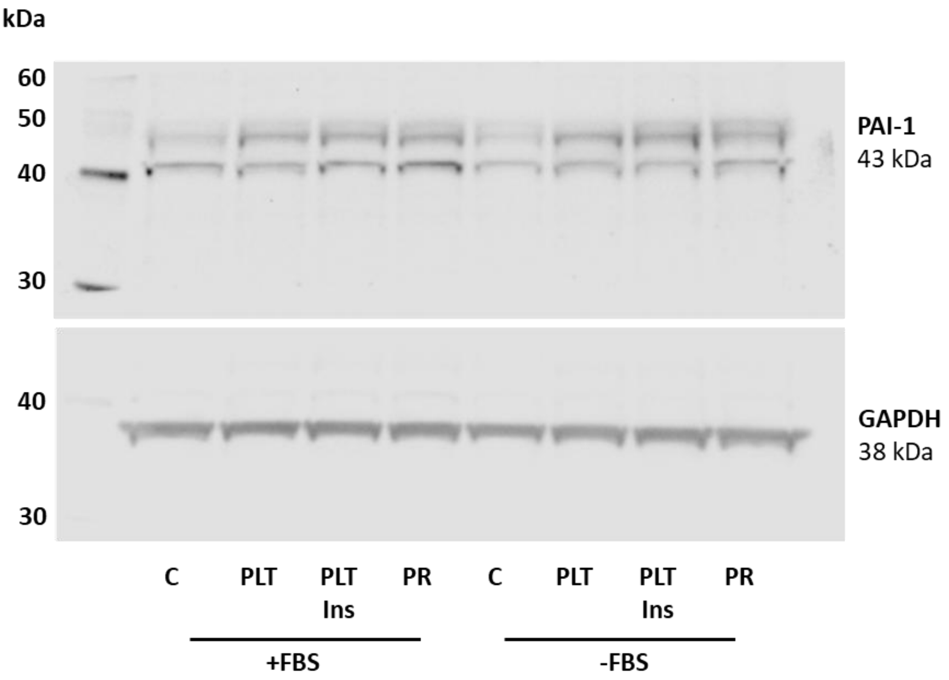

Supplementary Figure S10: Full sized image of western blot shown in Supplementary Figure 9C; C, Control; PLT, platelets directly on cells; PLT Ins, platelets in transwell inserts; PR, platelet releasate; FBS, fetal bovine serum.

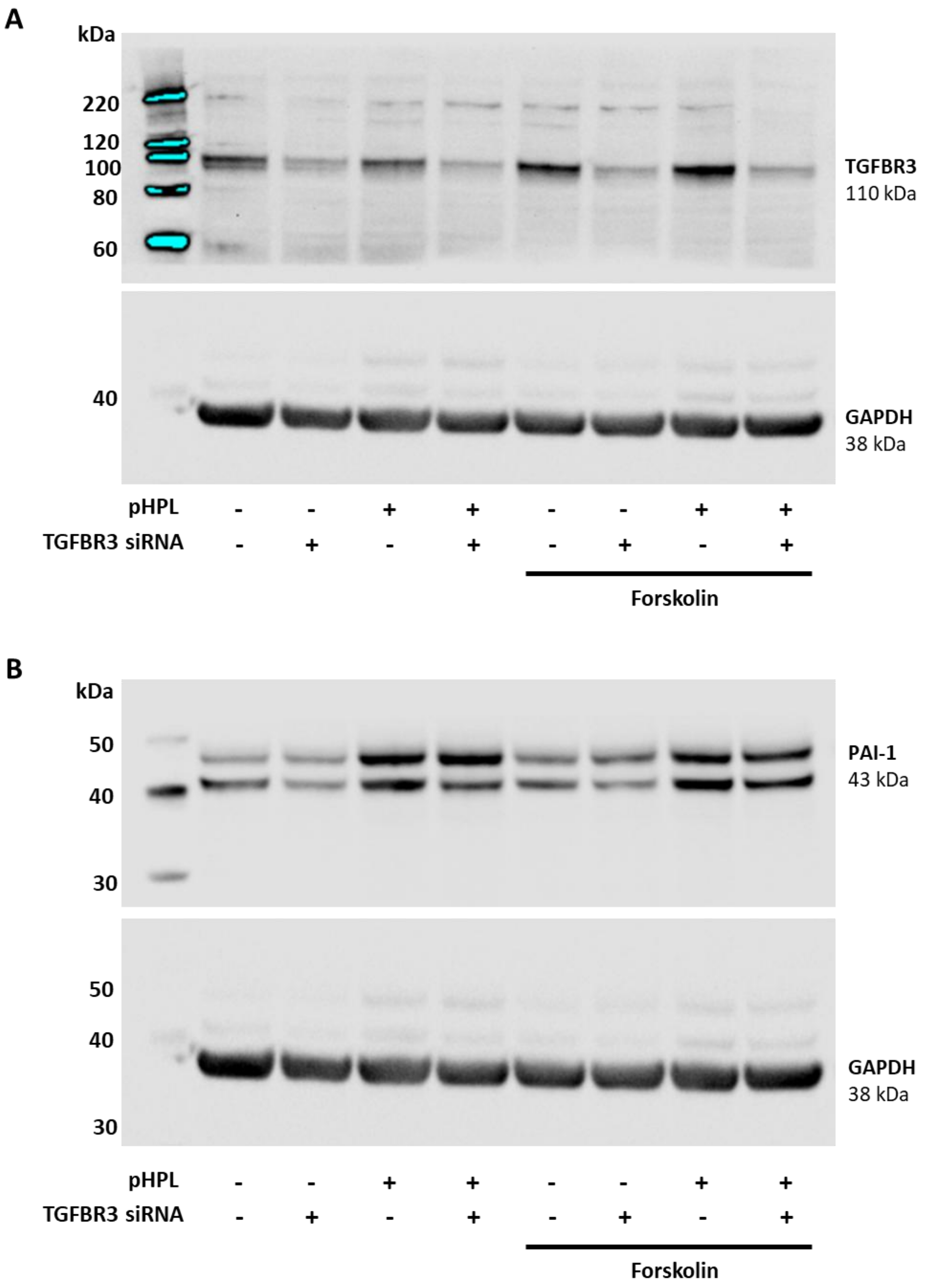

Supplementary Figure S11: Full sized image of western blot shown in Figure 6C+D; (**A**) is showing the full sized western blot image of Figure 6C and (**B**) is showing the full sized western blot image of Figure 6D. pHPL, pooled human platelet lysate; TGFBR3 siRNA, TGF-β type 3 receptor silencing RNA.

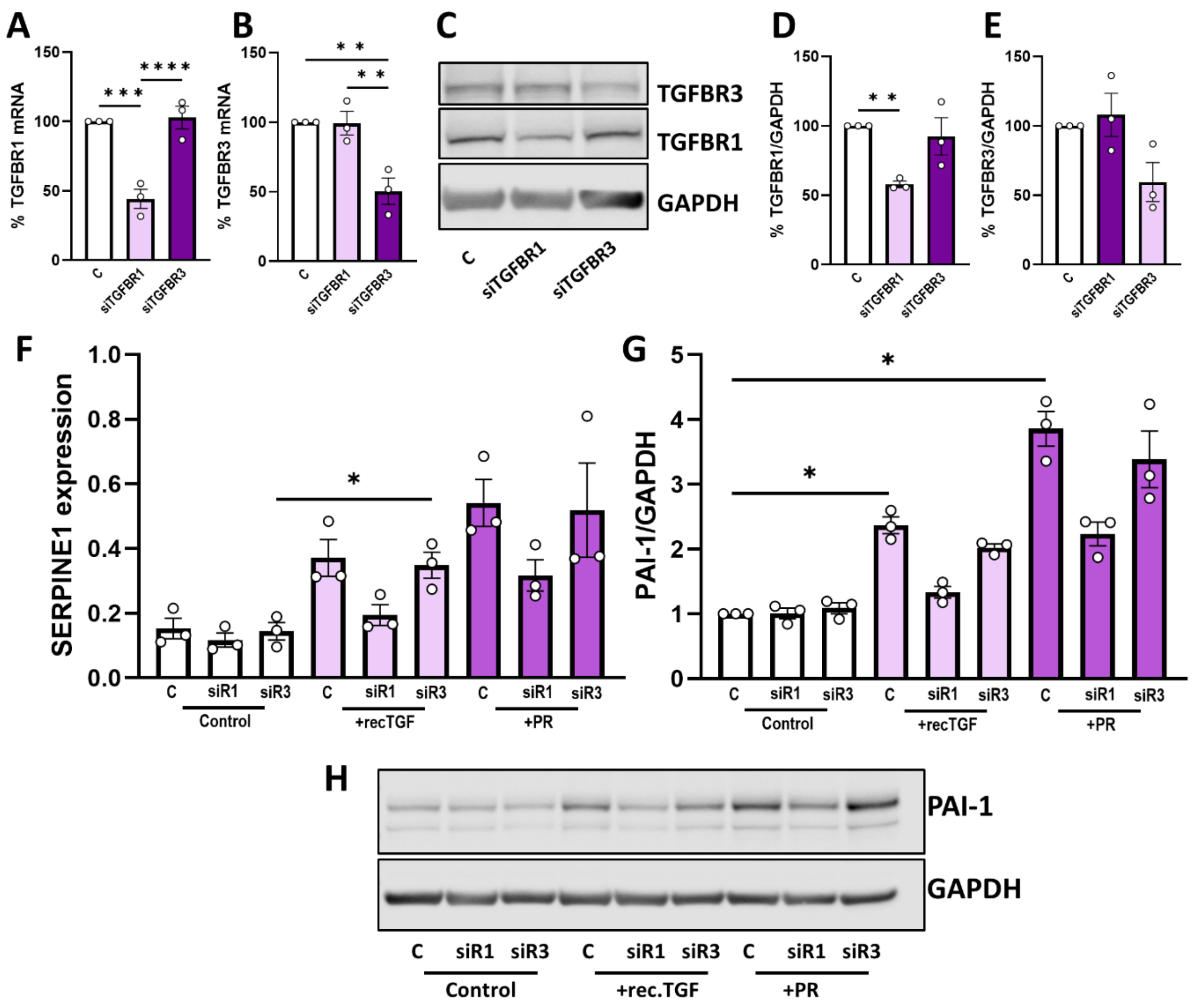

Supplementary Figure S12. *Upregulation of PAI-1 in response to TGF-β1 and platelet-derived factors is induced via TGFBR1*

BeWo cells were seeded in a 24-well plate with a density of 200.000 cells per well overnight before they were transfected with TGF-β type 1 receptor and TGF-β type 2 receptor Silencer® Select pre-designed siRNA (5 pmol; Ambion®, Thermo Fisher Scientific) for 24h (**A-H**). Gene expression analysis (**A** and **B**) as well as protein analysis (**C-E**) revealed a silencing efficiency of 50% of both, TGF-β type 1 receptor and TGF-β type 2 receptor compared to medium controls. (**F-H**) After silencing for 24h, cells were treated with either recombinant TGF-β1 (20 ng/ml) or platelet releasate for 24h. Recombinant TGF-β1- as well as platelet releasate-induced upregulation of *SERPINE1* (**F**) and PAI-1 (**G** and **H**) was attenuated when TGFBR1 was silenced. Data are represented as mean ± SEM from three independent experiments. (**A-H**) Significance was tested using a one-way ANOVA; $^*p \leq 0.05$, $^{**}p \leq 0.01$, $^{***}p \leq$

0.001; C, Control; siR1, siRNA TGF-β type 1 receptor; siR3, siRNA TGF-β type 3 receptor; TGFBR1, TGF-β type 1 receptor; TGFBR3, TGF-β type 3 receptor; rec.TGF, recombinant TGF-β1; PR, platelet releasate.

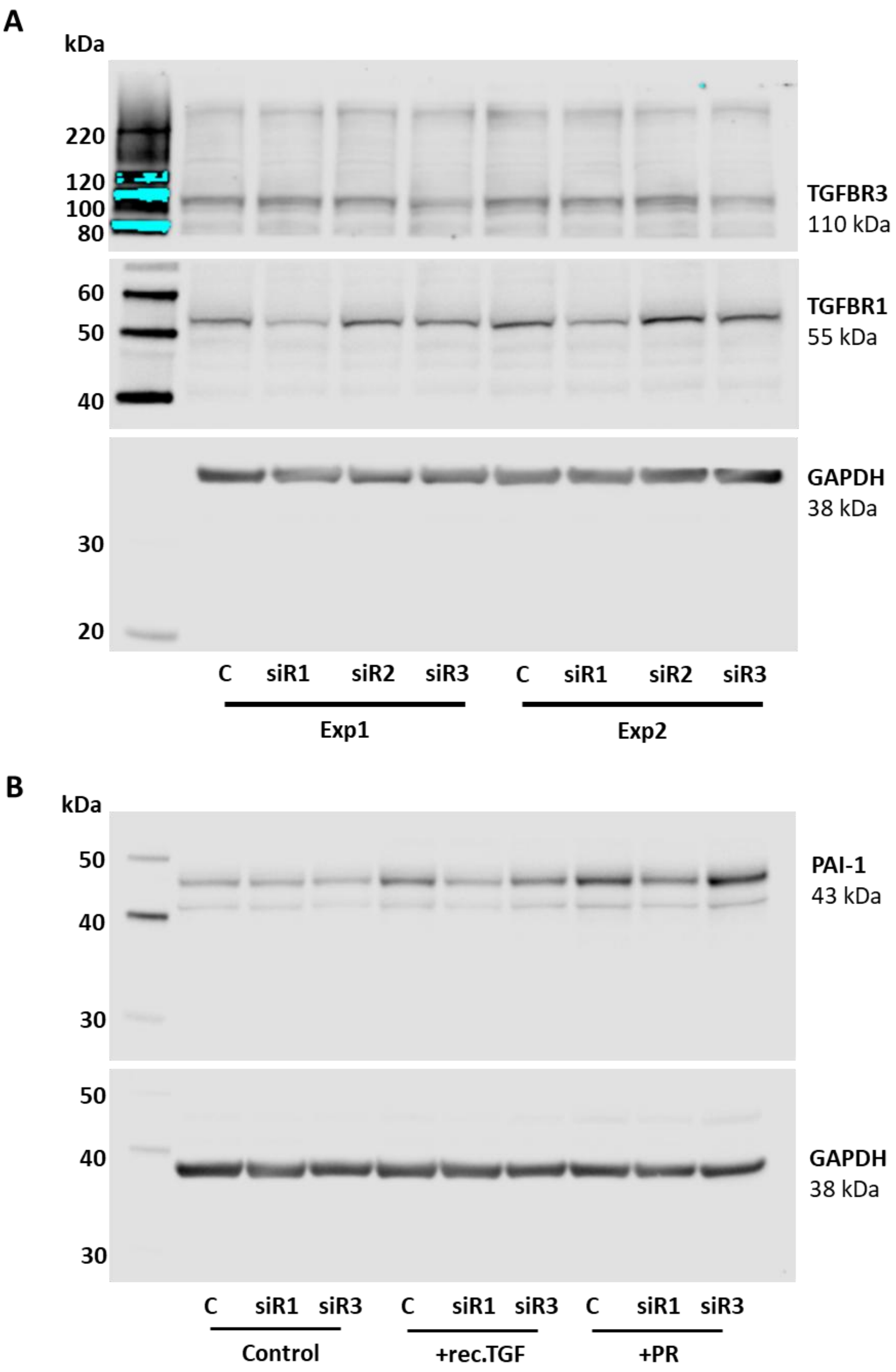

Supplementary Figure S13: Full sized image of western blot shown in Supplementary Figure 12C+H; (**A**) is showing the full sized western blot image of Supplementary Figure 12C and (**B**) is showing the full sized western blot image of Supplementary Figure 12H. C, control; siR1, TGF-β type 1 receptor silencing RNA; siR3, TGF-β type 3 receptor silencing RNA; rec.TGF, recombinant TGF-β1; PR, platelet releasate.

Supplementary Table S1. *Patient Characteristics of the study group shown in Figure 1*

		FT	early Ctrl	late Ctrl	loPE
		(n=42)	(n=5)	(n=31)	(n=23)
Maternal age	*years*	29.63 (7.45)	-	32.13 (5.08)	-
Maternal BMI (at birth)	*[kg/m^2]*	23.48 (4.76)	29.19 (9.30)	29.61 (5.23)	32.01 (7.87)
Maternal BMI (prepreg)	*[kg/m^2]*	-	23.99 (7.45)	24.50 (5.23)	26.03 (6.78)
Gestational age	*[weeks]*	8.53 (2.02)	31.83 (2.56)	38.05 (1.90)	36.15 (1.36)
Fetal weight	*[g]*	-	1894.00 (483.40)	3204.97 (648.86)	2423.09 (410.97)
Placental weight	*[g]*	-	430.00 (62.45)	596.00 (156.04)	529.52 (196.74)
Fetal sex	*f/m*	-	1/4	12/19	11/12
Smoker	*%*	43.33	-	-	-

Data are presented as mean (±SD); FT, first trimester; early ctrl = early (<34weeks) controls; late ctrl, late (≥34 weeks) controls; loPE, late-(≥34 weeks) onset preeclampsia; f, female; m, male; prepreg., before pregnancy.

Supplementary Table S2. *Patient Characteristics of the study group shown in Figure 2*

		FT	***Term***
		(n=12)	(n=5)
Maternal age	*years*	27.17 (5.15)	36.00 (5.83)
Maternal BMI (at birth)	*[kg/m²]*	22.57 (3.50)	27.29 (3.10)
Maternal BMI (prepreg)	*[kg/m²]*	-	21.68 (1.99)
Gestational age	*[weeks]*	7.86 (0.75)	38.49 (0.54)
Fetal weight	*[g]*	-	3350.00 (472.81)
Placental weight	*[g]*	-	628.00 (121.12)
Fetal sex	*f/m*	-	2/3
Smoker	*%*	58.33	0
Mean platelet count	*$x10^3/\mu l$*	162.25 (49.72)	179.25 (48.11)

Data are presented as mean (±SD); FT, first trimester; Term, third trimester; f, female; m, male; prepreg., before pregnancy.

Supplementary Table S3. *Patient Characteristics of the study group used for the preparation of platelet releasate*

		PR
		(n=67)
Maternal age	*years*	33.38 (5.38)
Maternal BMI (at birth)	*[kg/m²]*	26.53 (7.09)
Maternal BMI (prepreg)	*[kg/m²]*	23.87 (3.37)
Gestational age	*[weeks]*	38.50 (1.32)
Fetal weight	*[g]*	3231.04 (411.38)
Placental weight	*[g]*	584.00 (125.36)
Fetal sex	*f/m*	30/20
Smoker	*%*	11.760
Mean platelet count	*x10³/µl*	187.31 (63.39)

Data are presented as mean (±SD); PR, platelet releasate; f, female; m, male; prepreg., before pregnancy.

Supplementary Table S4. *Patient Characteristics of the study group used for isolation and characterization of platelet-derived extracellular vesicles in Figure 4 and Supplementary Figure 4*

		Non-pregnant	***Pregnant***
		(n=3)	(n=4)
Maternal/donor age	*years*	30.00 (3.46)	31.25 (1.50)
Maternal BMI (at birth)	*[kg/m²]*	-	31.63 (6.95)
Maternal BMI (prepreg)	*[kg/m²]*	20.36 (0.85)	27.38 (8.67)
Gestational age	*[weeks]*	-	38.89 (0.29)
Fetal weight	*[g]*	-	3052.50 (231.14)
Placental weight	*[g]*	-	502.50 (94.65)
Fetal sex	*f/m*	-	1/3
Smoker	*%*	0	0

Data are presented as mean (±SD); f, female; m, male; prepreg., before pregnancy.

Supplementary Table S5. *Forward and reverse primer sequences for gene expression analysis using qPCR.*

**Gene**	**forward (5'–3')**	**reverse (5'–3')**
SERPINE1	GTTCTGCCCAAGTTCTCCCT	ACATGTCGGTCATTCCCAGG
TGFBR3	TTGCCTAAGTGTGTGCCTCC	CACAGCAAGGGGCTTAGTGA
YWHAZ	GGTGGCCAATATGGGGATGT	TCCCTTTTATTCCCCGCCAG
GAPDH	ACCCACTCCTCCACCTTTGA	CTGTTGCTGTAGCCAAATTCG
TBP	TGA CCC AGC ATC ACT GTT TC	CCA GCA CAC TCT TCT CAG CA
TGFBR1	AGCAGCAGACAATAAAGACAATGG	CCGTGGACAGAGCAAGTTTT

Supplementary Table S6. *Primary and secondary antibodies for western blot.*

	Species	***Clone***	***Company***	***kDa***	***Western Blot***
β-Actin	monoclonal mouse	C-2	Santa Cruz Biotechnology, Inc., Dallas, TX, USA	43	1:500000 0.4 ng/ml
GAPDH	monoclonal rabbit	14C10	Cell Signaling Technology®, Danvers, MA, USA	37	1:5000
PAI-1	monoclonal rabbit	EPR17272-21	Abcam, Cambridge, UK	45	1:1000 0.674 µg/ml
TGFBR3	polyclonal rabbit	/	Cell Signaling Technology®, Danvers, MA, USA	110	1:1000
TGFBR1	monoclonal rabbit	EPR20923-13	Abcam, Cambridge, UK	55	1:1000 0.542 mg/ml
Vinculin	polyclonal rabbit	/	Cell Signaling Technology®, Danvers, MA, USA	124	1:1000
goat anti-mouse	goat, HRP-conjugated	/	Bio-Rad Laboratories, Hercules, CA, USA		1:3000
goat anti-rabbit	goat, HRP-conjugated	/	Bio-Rad Laboratories, Hercules, CA, USA		1:5000

The final concentration (if known) is stated below the used dilution factor.

Supplementary Table S7. *Primary and secondary antibodies for immunofluorescence and immunohistochemistry*

	Primary antibody				
	Species	***Clone***	***Company***	***IHC***	***IF***
Fibrin	monoclonal mouse	59D8	Merck KGaA, Darmstadt, Germany		1:500
ß-hCG	polyclonal rabbit	/	Proteintech®, Rosemont, IL, USA		1:100
CD42b	polyclonal rabbit	/	Proteintech®, Rosemont, IL, USA	1:1000	1:500
PAI-1	monoclonal mouse	1D5	Abcam, Cambridge, UK	1:2000	
CK7	monoclonal rabbit	R17-S	Bio-Techne, Minneapolis, MN, USA		1:100
	Secondary antibody				
	Species	***Clone***	***Company***	***IHC***	***IF***
Alexa Fluor® 555	goat-anti-mouse IgG (H+L)	/	Invitrogen™ Corporation, Thermo Fisher Scientific, Carlsbad, CA, USA		1:200 10 µg/ml
Alexa Fluor® 633	goat-anti-rabbit IgG (H+L)	/	Invitrogen™ Corporation, Thermo Fisher Scientific, Carlsbad, CA, USA		1:200 10 µg/ml
Alexa Fluor® 488	goat-anti-mouse IgG (H+L)	/	Invitrogen™ Corporation, Thermo Fisher Scientific, Carlsbad, CA, USA		1:200 10 µg/ml

The final concentration (if known) is stated below the used dilution factor.

Supplementary Table S8. *Patient Characteristics of the study group used for Supplementary Figure 1*

		**Control**	**PE**
		(n=11)	(n=11)
Maternal BMI (at birth)	*[kg/m^2]*	33.06 (6.43)	33.99 (9.37)
Maternal BMI (prepreg)	*[kg/m^2]*	26.44 (6.61)	28.61 (8.87)
Gestational age	*[weeks]*	37.70 (2.60)	35.06 (2.44)
Fetal weight	*[g]*	2966.64 (790.92)	2057.82 (601.16)
Placental weight	*[g]*	548.18 (183.51)	414.55 (116.74)
Fetal sex	*f/m*	3/8	4/7

Data are presented as mean (±SD); PE, preeclampsia; f, female; m, male; prepreg., before pregnancy.

Supplementary Materials and Methods

Platelet isolation

Platelet counts of whole citrated blood samples were measured by the Sysmex KX-21 N Automated Hematology Analyzer (Sysmex, Illinois, IL, USA). After generation of platelet rich plasma (PRP) by centrifugation of whole blood samples at 100 *g* for 15 min at RT, PRP was gently mixed to a total volume of about 20 ml with platelet wash buffer, consisting of aqua dest. with 128 mM NaCl (Supelco®, Merck; Darmstadt, Germany), 11 mM Glucose (Sigma Aldrich, St. Louis, MO, USA) 7.5 mM Na2HPO4 (Merck), 4.8 mM sodium citrate (Sigma Aldrich), 4.3 mM NaH2PO4 (Lactan; Graz, Austria), 2.4 mM citric acid (Merck) and 0.35% bovine serum albumin (Biowest; Nuaillé, France). In order to avoid platelet activation during isolation, 2.5 ng/µL of prostaglandin (Cayman Chemical Company; Ann Arbor, MI, USA) were added. After another centrifugation step at 1962g for 15 min at RT, the platelet pellet was resuspended in 7 mL platelet wash buffer and centrifuged again at 1962g for 15 min at RT. The supernatant was gently removed and the pelleted platelets were resuspended in serum-free DMEM/F12 (1:1, Gibco™, Thermo Fisher Scientific, Waltham, USA) supplemented with 0.1 U/mL Penicillin/Streptomycin (Gibco™, Thermo Fisher Scientific) and 1% L-glutamine (Gibco™, Thermo Fisher Scientific) in the initial plasma volume. The washed platelets were then either used for co-cultures with placental villi and trophoblast cells or further processed for the preparation of platelet releasate.

Culture of trophoblast cell line BeWo

The trophoblast cell line BeWo, which was purchased from the European Collection of Cell Cultures (ECACC), was used for *in vitro* studies. Cells were cultured with a density of 1 x 10^5

cells/ml in 24- or 12-well culture dishes (Nunc Lab-Tek, Thermo Fisher; NY, USA) in DMEM/ F12 (1:1, Gibco™, Thermo Fisher Scientific) supplemented with 10% FBS (Gibco™, Thermo Fisher Scientific), 0.1 U/mL Penicillin/Streptomycin (Gibco™) and 1%
L-glutamine (Gibco™). After an overnight incubation in a humidified atmosphere of 5% CO_2 at 37°C, treatment was applied as indicated.

Preparation of protein lysates

BeWo cells and tissue samples were washed in PBS, afterwards lysed in RIPA buffer (Sigma-Aldrich, St. Louis, USA), supplemented with protease inhibitor cocktail (Roche Diagnostics; Mannheim, Germany) and PhosSTOP (Roche Diagnostics, Mannheim, Germany). Placental tissue samples were further homogenized with the TissueLyser LT (Qiagen, Hilden, Germany) and Stainless Steel Beads (5 mm, Qiagen, Hilden, Germany) and subsequently sonified with a Bioruptor® Pico sonication device (Diagenode, Liège, Belgium) for 10 cycles at 4°C. After centrifugation at 8,000 g and 4°C for 10 min, total protein concentration was determined in clear supernatants according to Lowry method.

Recombinant PAI-1

A stock solution of recombinant PAI-1 (Merck; Darmstadt, Germany) at a concentration of 250 ng/µl was diluted with aqua dest. in a series of dilutions ranging from 250 ng to 1.95 ng. The solution was then subjected to immunoblotting for PAI-1.

Immunohistochemistry

For immunohistochemical staining, 5 µm thick human formalin-fixed paraffin-embedded (FFPE) sections from placenta tissue from the first and third trimester was mounted on

Superfrost Plus slides (Menzel-Gläser, Thermo Scientific, Waltham, USA) and afterwards deparaffinized. For standard antigen retrieval, sections were placed in an Epitope Retrieval Solution (pH 9.0; Novocostra, Leica, Wetzlar, Germany) or citrated buffer (pH 6.0) for two 20-minute periods at 150 W in a laboratory microwave (Miele; Guetersloh, Germany). For immunohistochemistry, the UltraVision Large Volume Detection System HRP Polymer Kit (Thermo Fisher Scientific, Waltham, MA, USA) was used as previously described (Blaschitz *et al.*, 2015), and afterwards nuclei were stained with Mayer's hemalaun (Thermo Fisher Scientific, Waltham, MA, USA). Slides were mounted with Kaiser's glycerol gelatine (Merck). For negative controls, adjacent slides from serial sections were incubated with Negative Control for Rabbit Immunoglobulin Fraction (1:7500; Dako, Agilent Technologies, Santa Clara, USA). For immunofluorescence double staining, slides were stained as previously described (Lyssy *et al.*, 2025). In brief sections were blocked with Ultra V block (Thermo Fisher Scientific, Waltham, MA, USA) before incubation with primary antibodies for 30 minutes. Slides were washed three times, before secondary fluorescence-labelled antibodies were applied for another 30 minutes. After staining the nuclei with DAPI (1:2000 in PBS), slides were mounted with ProLong Gold Antifade reagent (Invitrogen^{TM} Corporation, Thermo Fisher Scientific, Carlsbad, CA, USA). All images were obtained with an SLIDEVIEW^{TM} VS200 Slide Scanner (Evident Europe GmbH, Hamburg, Germany). Used antibodies are summarized in Supplementary Table S7.

Software-based quantification of fibrin

Placental tissue from first and third trimester was either fluorescence double-stained for fibrin and the platelet marker Cd42b or immunohistochemical stained for Cd42b, as previously described. Fluorescence images of the whole section were obtained using an Evident Olympus

VS200 Slide Scanner and images were afterwards subjected to Visiopharm (Version 2021.09, Hørsholm, Denmark) for a software-based quantification of fibrin. To ensure the detection of floating villi exclusively, decidual structures and anchoring villi, were manually excluded from the quantification area. Villous regions were detected within each sample by applying a threshold on a feature combining a polynomial gradient filter of FITC and a mean filter feature of the DAPI channel. Fibrin within the villous regions was detected by a threshold within the FITC channel. Measured Fibrin area (µm^2) was normalized to total villi area (µm^2).

The immunohistochemical images were acquired using an Olympus BX63 microscope (Olympus Corporation, Tokyo, Japan) and analyzed using ilastik (Version 1.3.3, EMBL, Heidelberg, Germany) and CellProfiler (Version 3.1.9, Broad Institute, Cambridge, MA, USA). Ilastik (EMBL) was used to classify the images into background (intervillous space), trophoblasts, erythrocytes, CD42b-positive platelets and stroma area using a pixel classification trained on an image subset. The classified images were then further analyzed using CellProfiler (Broad Institute), to measure CD42b-positive platelets in relation to the villous surface. Detected villous surface regions were segmented and masked by the CD42b-positive stained platelets obtained from ilastik (EMBL), allowing a quantification of CD42b-positive villous surface, representing adherent platelets, in relation to the total villous surface.

References

Blaschitz A, Siwetz M, Schlenke P, Gauster M. Adhering maternal platelets can contribute to the cytokine and chemokine cocktail released by human first trimester villous placenta. *Placenta* 2015;**36**:1333–1336.

Lyssy F, Forstner D, Guettler J, Kupper N, Ujčič K, Neuper L, Daxboeck C, El-Heliebi A, Kummer D, Krappinger JC, *et al.* Maternal platelet-derived factors induce trophoblastic LAIR2 expression to promote trophoblast invasion and inhibit platelet activation at the fetal-maternal interface. *J Thromb Haemost JTH* 2025;**23**:2010–2024.
